# Iron Coordination Properties of Gramibactin as Model for the New Class of Diazeniumdiolate Based Siderophores

**DOI:** 10.1002/chem.202003842

**Published:** 2021-01-14

**Authors:** Sofia Gama, Ron Hermenau, Mariachiara Frontauria, Demetrio Milea, Silvio Sammartano, Christian Hertweck, Winfried Plass

**Affiliations:** ^1^ Institut für Anorganische und Analytische Chemie, Friedrich-Schiller-Universität Jena Humboldtstr 8 07743 Jena Germany; ^2^ New address: Department of Analytical Chemistry Faculty of Chemistry University of Bialystok Ciolkowskiego 1K, 15–245 Bialystok Poland; ^3^ Department of Biomolecular Chemistry Leibniz Institute for Natural Product Research and Infection Biology (HKI) Beutenbergstr 11a 07745 Jena Germany; ^4^ Dipartimento di Scienze Chimiche, Biologiche, Farmaceutiche ed Ambientali Università degli Studi di Messina V.le F. Stagno d'Alcontres, 31 98166 Messina Italy; ^5^ Faculty of Biological Sciences Friedrich Schiller University Jena 07743 Jena Germany

**Keywords:** chemical speciation, diazeniumdiolate, iron(III) coordination, sequestering ability, siderophores

## Abstract

Gramibactin (GBT) is an archetype for the new class of diazeniumdiolate siderophores, produced by *Paraburkholderia graminis*, a cereal‐associated rhizosphere bacterium, for which a detailed solution thermodynamic study exploring the iron coordination properties is reported. The acid‐base behavior of gramibactin as well as its complexing ability toward Fe^3+^ was studied over a wide range of pH values (2≤pH≤11). For the latter the ligand‐competition method employing EDTA was used. Only two species are formed: [Fe(GBT)]^−^ (pH 2 to 9) and [Fe(GBT)(OH)_2_]^3−^ (pH≥9). The formation of [Fe(GBT)]^−^ and its occurrence in real systems was confirmed by LC‐HRESIMS analysis of the bacteria culture broth extract. The sequestering ability of gramibactin was also evaluated in terms of the parameters pFe and pL_0.5_. Gramibactin exhibits a higher sequestering ability toward Fe^3+^ than EDTA and of the same order of magnitude as hydroxamate‐type microbial siderophores, but smaller than most of the catecholate‐type siderophores and much higher than the most known phytosiderophores.

## Graphical abstract

**The sequestration properties** of gramibactin as a prototype for the new class of diazeniumdiolate siderophores is presented and compared with siderophores derived from catecholates and hydroxamates. According to pFe and pL_0.5_ values, gramibactin is a considerably more potent sequestering agent than the known class of phytosiderophores and approximately reaches the ability of typical hydroxamates like desferrioxamines.

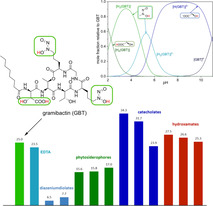

## Introduction

**Scheme 1:**
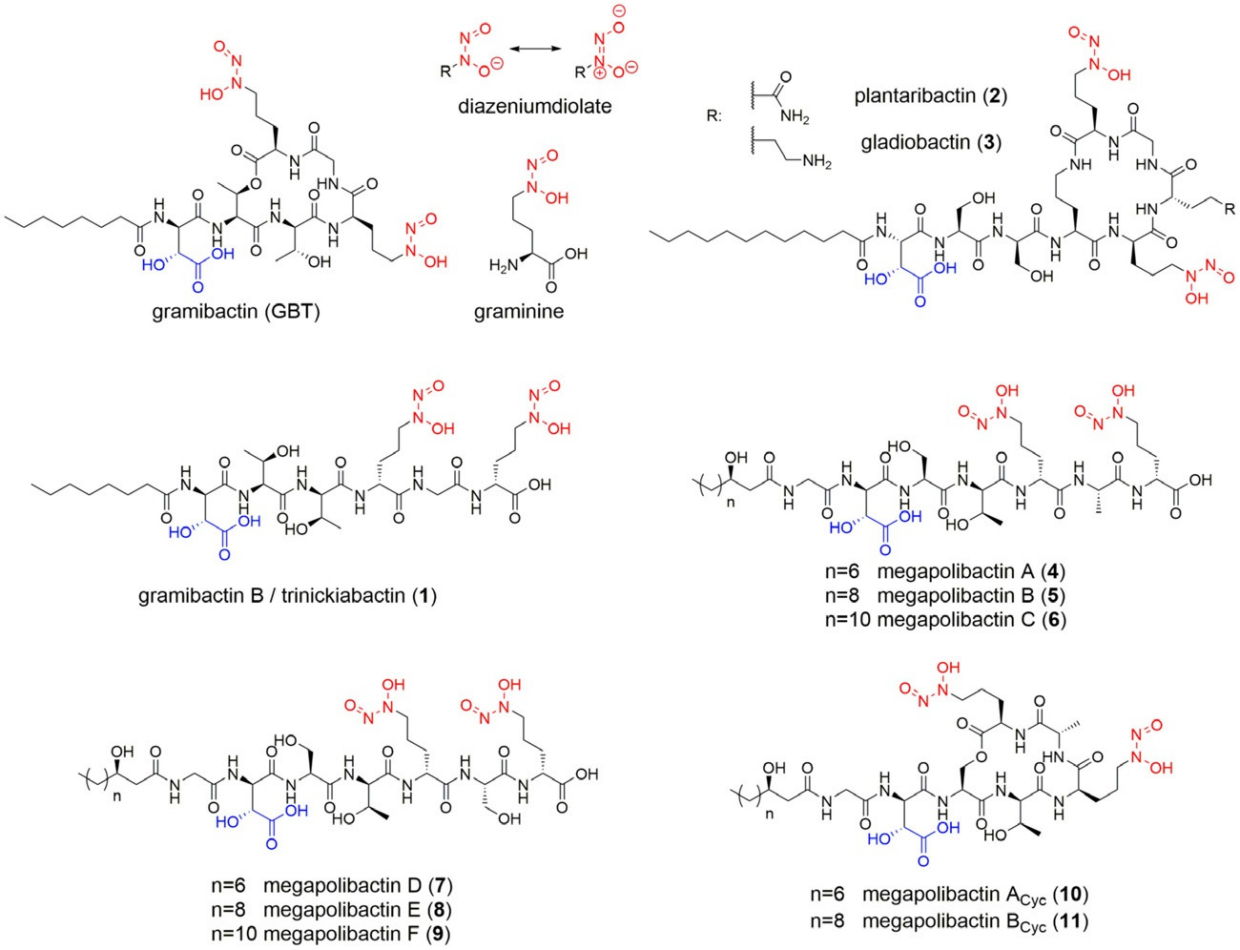
Gramibactin as a prototype of the new class of diazeniumdiolate siderophores containing graminine with relevant chelating sites: *N*‐nitroso‐*N*‐hydroxylamine group (red) and α‐hydroxocarboxylic acid (blue).

In the ongoing study of the mechanisms used by microorganisms and plants to cope with the limited supply of soluble iron, it has been found that bacteria can play a key role in the rhizosphere, as their siderophores may solubilize iron and make it accessible to the host plant.^[1]^ Along these investigations, gramibactin (GBT, Scheme 1) was recently identified as a novel siderophore produced by *Paraburkholderia graminis*, a bacterium isolated from the rhizosphere of maize and wheat plants.^[2]^ In this context it has been demonstrated that iron‐gramibactin metal complexes represent a viable source of iron for maize (*Zea mays* L. ssp. saccharata) plants.^[2]^

Siderophores are small organic molecules produced by microorganisms (microbial siderophores) or by graminaceous plants (phytosiderophores), characterized by a high affinity toward complexation of ferric ions. Microbial siderophores are good iron sequestering agents mainly due to strong iron chelating groups as hydroxamates, catecholates and/or α‐hydroxocarboxylates, while phytosiderophores, such as mugineic acid and its derivatives (e.g., deoxymugineic acid, epi‐hydroxymugineic acid, nicotianamine, Scheme S1), are polydentate ligands with amine and carboxylate groups as metal binding sites.[^3^, ^4^, ^5^, ^6^] However, with the identification of gramibactin, the diazeniumdiolate (*N*‐nitroso‐*N*‐hydroxylamine) group (Scheme 1, red) should now also be included in the list of typical chelating moieties present in siderophores. Gramibactin is a cyclopeptide that contains a classical α‐hydroxocarboxylate moiety (Scheme 1, blue) in combination with two *N*‐nitroso‐*N*‐hydroxylamine groups (Scheme 1, red), introduced by the non‐canonical amino acid d‐graminine. Moreover, genome mining revealed in addition to the acyclic form of gramibactin (gramibactin B/trinickiabactin^[7]^ −**1**) two new types of plant‐associated diazeniumdiolate‐based bacterial siderophores,^[8]^ namely plantaribactin (**2**) and its congener gladiobactin (**3**) as well as the family of megapolibactins (**4**–**11**).

Despite these seminal discoveries, the presence of the *N*‐nitroso‐*N*‐hydroxylamine group has already been reported in other natural products (some examples are reported in Table S1).[^9^, ^10^, ^11^, ^12^, ^13^, ^14^, ^15^] Interestingly, several of the reported diazeniumdiolates present high biological activity, as antibacterial, antiviral, antifungal or even antitumoral agents, which seems to be directly related to the chelating ability of these compounds.[^9^, ^12^, ^13^, ^14^, ^15^, ^16^, ^17^, ^18^, ^19^, ^20^] This is the case with fragin, for example, for which C. Jenul et al. have demonstrated that metal chelation is the molecular basis for its observed antifungal activity.^[15]^ Likewise, the action of dopastin as a dopamine β‐hydroxylase inhibitor is due to its interaction with the copper ion present in the active center of the enzyme.^[12]^ In addition, nitrosoxacins inhibit 5‐lipoxygenase, an iron‐metalloenzyme that is involved in the biosynthesis of lipid mediators in inflammation processes.[^13^, ^14^, ^20^] The observed strong dependence between biological activity and metal ion interactions for *N*‐nitroso‐*N*‐hydroxylamine compounds along with the recent discovery of gramibactin acting as a siderophore generates a fundamental interest in its complexation properties. Furthermore, providing relevant benchmarks for chelation will set the foundations for future interest in *N*‐nitroso‐*N*‐hydroxylamine derivatives as starting points for the development of new compounds with potential pharmaceutical application. Remarkably enough, to our knowledge, the binding ability and/or the chemical speciation of bacterial/natural *N*‐nitroso‐*N*‐hydroxylamine compounds toward metal ions as well as the stability of their complexes have never been investigated in detail.

In this work, we used a thermodynamic approach to gain insight into the details of iron sequestration by gramibactin. We aim to contribute to a better comprehension of the iron acquisition and uptake mechanisms and the role of this *N*‐nitroso‐*N*‐hydroxylamine group in siderophore. Along this line, the complete characterization of the chelating ability of gramibactin toward iron(III) in aqueous solution over a wide pH range (2.0≤pH≤11.0) is reported. The studies were carried out by potentiometric and spectrophotometric titrations in aqueous KCl solution and the presence of the relevant iron‐gramibactin species was confirmed by mass spectrometry.

The thermodynamic data obtained here establish the basis for a detailed assessment of gramibactin as bacterial siderophore, especially in comparison to other well‐known classes. This assessment is carried out on the basis of sequestration parameters such as pL_0.5_ and pM, with the latter being well known among a large scientific community from different fields. Since these parameters, in particular pM, are often used by researchers that are not regularly involved in their determination or who are not always aware of the correct conditions to be considered in their calculation process, we herein report a detailed explanation of how and in which conditions they should be determined. With the presented data and details in this paper we aim to promote the correct use and evaluation of speciation parameters and to avoid future misleading results and comparisons. This description is accompanied with an exhaustive determination of both parameters for relevant compounds such as different diazeniumdiolates, phytosiderophores, and relevant siderophores, as well as EDTA, a commonly used chelating agent in biological systems.

## Results and Discussion

### Acid‐base properties of gramibactin

The acid‐base behavior of gramibactin was studied and protonation constants were determined experimentally by systematic potentiometric titrations under defined conditions of temperature, medium, and ionic strength (i.e., *I*=0.1 mol dm^−3^ in KCl_(aq)_ and *T=*298.15±0.1 K).

Gramibactin carries several functional groups that can be involved in various protonation/deprotonation equilibria, namely two hydroxyl groups, one of which together with a carboxylic group is part of an α‐hydroxocarboxylate moiety, and two *N*‐nitroso‐*N*‐hydroxylamine groups. Four protonation steps can be observed within the investigated pH range (2≤pH≤11) and the corresponding protonation constants are summarized in Table 1.

**Table 1:** Protonation constants of gramibactin (GBT) in KCl_(aq)_ at *I*=0.1 mol dm^−3^ and *T=*298.15 K.

Species	*q*:*r*	log *β*_qr_^[a]^	log *K*_1_ r ^[b]^
[H(GBT)]^3−^	1:1	10.94±0.02	
[H_2_(GBT)]^2−^	1:2	16.65±0.01	5.71
[H_3_(GBT)]^−^	1:3	21.52±0.01	4.87
[H_4_(GBT)]	1:4	23.79±0.02	2.27

[a] log*β*_qr_ values refer to the equilibrium: *q* L+*r* H=L_*q*_H_*r*_ ±95 % confidence interval; ^[b]^ log *K*_1r_ values refer to the equilibrium: H+LH_*r*−1_⇄LH_*r*_.

**Figure 1:**
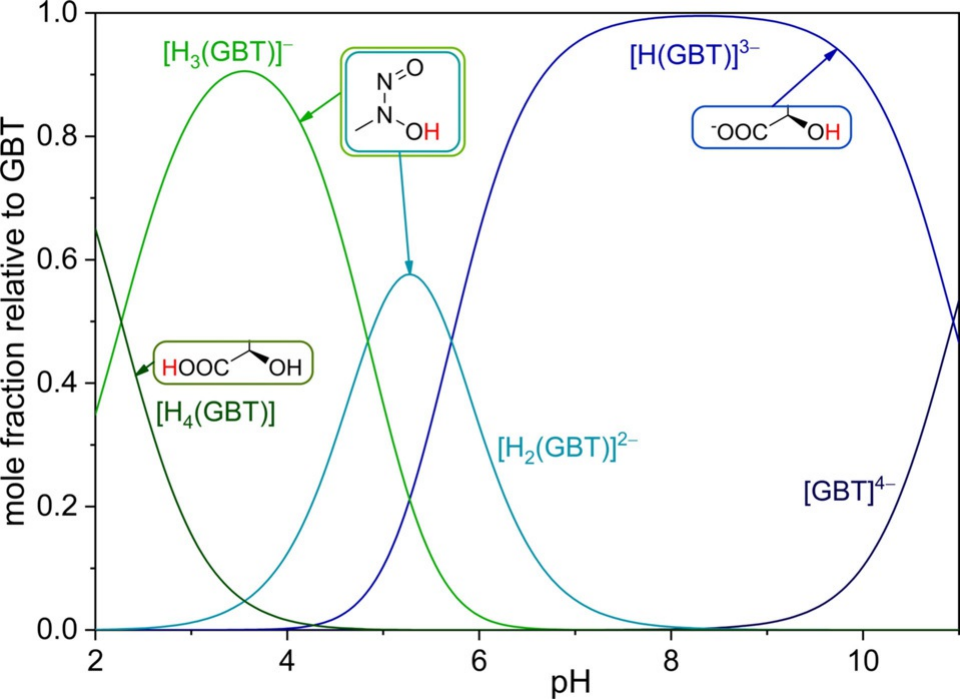
Distribution diagram for the protonated [H_r_(GBT)]^r−4^ species of gramibactin as a function of pH value in KCl_(aq)_ at *I*=0.1 mol dm^−3^ and at *T=*298.15 K with *c*_GBT_=1 mmol dm^−3^. Structural schemes indicate the protons (red) involved in the acid‐base equilibrium of the corresponding species.

Given the molecular structure and based on the reference of similar systems reported in several thermodynamic databases,[^21^, ^22^, ^23^] the carboxylic group can be expected as the last to be protonated, with an observed log *K* value of 2.27, whereas the hydroxyl groups should be protonated first. In the case of gramibactin, only the protonation of the hydroxyl group of the α‐hydroxocarboxylate moiety could be determined (log *K=*10.94), which nicely corresponds to literature values reported for Rhizoferrin (11.3 and 10.05) that also contains α‐hydroxocarboxylate moieties.^[24]^ The deprotonation of the second hydroxyl group which is not acidified by neighboring functional groups is expected to occur at pH values greater than 11, which is above the investigated pH range. Consistent with reported protonation constants for reference compounds containing the *N*‐nitroso‐*N*‐hydroxylamine moiety, such as dopastin (log *K=*5.2)^[12]^ and nitrosofungin (log *K=*5.1)[^12^, ^13^] (see Table S1 for structures), the processes observed at log *K* values of 5.71 and 4.87 can be assigned to the protonation of the two diazeniumdiolate groups (cf. Scheme S2). The distribution diagram of the gramibactin species with varying protonation state based on the determined protonation constants is depicted in Figure 1.

The monoprotonated gramibactin [H(GBT)]^3−^ is the major species at neutral to basic pH values (7<pH<9), while the fully deprotonated species [GBT]^4−^ starts to form at pH values above 9. The tri‐ and tetraprotonated gramibactin species mainly occur at pH values below 5. The fully protonated, neutral gramibactin [H_4_(GBT)] only exists at very acidic pH values (with ca. 20 % at pH≈3). From these results, it is evident that gramibactin is mainly present as a negatively charged compound in the soil microenvironment, not only within the normal pH range used for culturing plants (4≤pH≤10.5),^[25]^ but also in acidic soils (pH≤5.5).^[26]^

**Figure 2:**
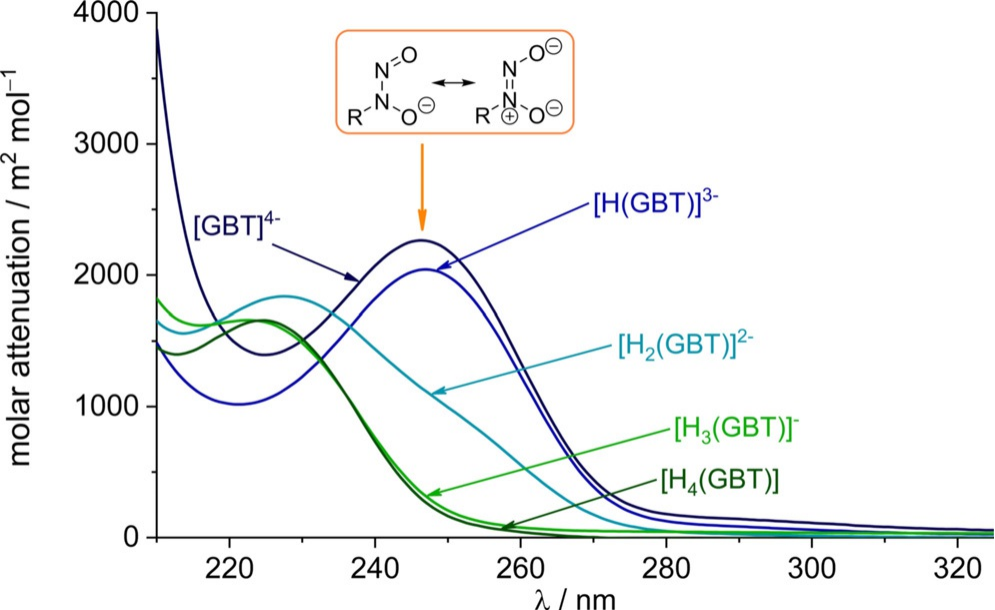
Calculated molar absorbance spectra for the individual protonated gramibactin species [H_r_(GBT)]^*r*−4^ (*r*=0–4).

The data obtained independently by spectrophotometric titrations not only confirmed the protonation constants derived from potentiometric titrations (see Table 1), but also allowed the spectrophotometric characterization of the individual species. Actually, the molar attenuation of all gramibactin species [H_r_(GBT)]^r−4^ (*r*=0–4) could be determined from fitting the experimental data on the basis of the given equilibrium model using the HypSpec program.^[27]^ The corresponding spectra are depicted in Figure 2.

The spectrum of the fully protonated form of gramibactin [H_4_(GBT)] is characterized by a band with *λ*_max_ at 225 nm (*ϵ*≅1650 m^2^ mol^−1^). As anticipated, the spectrum is virtually unchanged for the mono deprotonated form [H_3_(GBT)]^−^, since this is related to the deprotonation of the carboxylic proton of the α‐hydroxocarboxylate moiety. However, for the doubly deprotonated species [H_2_(GBT)]^2−^ a slight bathochromic shift of the former band and the appearance of an additional shoulder at about 250 nm is observed. For the triply deprotonated species [H(GBT)]^3−^ the former band at 225 nm disappears and the previous shoulder now appears as an intense band at 246 nm. This observation is consistent with the assignment of the latter two (second and third) deprotonation steps to the *N*‐hydroxylamine protons. The fourth deprotonation step assigned to the proton of the hydroxyl group of the α‐hydroxocarboxylate moiety leaves the spectrum for the corresponding species [(GBT)]^4−^ virtually unchanged (*λ*_max_=246 nm, *ϵ*≅2260 m^2^ mol^−1^).

The observed behavior can be rationalized based on the literature‐known protonation‐associated variation of the optical properties of the *N*‐nitroso‐*N*‐hydroxylamine group. The protonated form is characterized by an intense, broad asymmetric UV absorption band at about 229 to 232 nm, which is assigned to a π–π* transition, whereas upon deprotonation this absorption band undergoes a bathochromic shift and appears in the range from 244 to 258 nm with a slight increase in the molar attenuation coefficient.[^19^, ^28^, ^29^, ^30^, ^31^] Consequently, the present spectra support the deprotonation equilibria depicted in Scheme S2.

### Speciation of the iron(III)‐gramibactin system

For the determination of the stability constants of the iron(III)‐gramibactin species, two important aspects need to be considered for an adequate description of the system.

At first, this concerns the sole speciation of iron(III) in aqueous solution, that is, the iron(III) hydrolysis. In fact, for strong Lewis acids such as iron(III), the acid‐base properties (i.e. hydrolysis) must be taken into account for a correct speciation model of the system under investigation. For this work, we considered the mononuclear [Fe(OH)]^2+^, [Fe(OH)_2_]^+^, [Fe(OH)_3_], and [Fe(OH)_4_]^−^ as well as the polynuclear [Fe_2_(OH)_2_]^4+^ and [Fe_3_(OH)_4_]^5+^ species, since they seem to be the most reliable and appropriate to describe iron(III) speciation at various concentration levels.[^32^, ^33^, ^34^] As the formation of the polynuclear species [Fe_12_(OH)_34_]^2+^ could also be relevant even at low concentrations,[^21^, ^23^, ^32^, ^33^, ^34^] the effect of its inclusion/exclusion in the speciation model of our system was investigated by performing the calculations in both ways. The corresponding values used in this work were adapted from refs.[^32^, ^33^, ^34^] and are summarized in Table S2 of the Supporting Information.

The second aspect, that needs to be considered here, becomes important for ligands with a strong chelating ability toward the metal ion under investigation. For iron(III)‐siderophore systems, formation constants higher than 10^15^–10^20^ can be expected, which results in an almost complete shift of the formation equilibrium given in Equation 1 toward the product side.

(1) $$M+\mathrm{LH}{}_{r}\overset{\leftarrow \;\;}{⟶}\mathrm{ML}+r\;H$$

In such cases, the classical procedure to determine stability constants by proton displacement experiments (acid‐base titrations by exploiting the above‐reported equilibrium) utilizing the reaction of the ligand with the relevant metal ion cannot be performed properly, since practically solely the product complex is present as soon as metal ion and ligand solutions are mixed. A suitable strategy to overcome this problem is to apply the ligand‐competition method.[^35^, ^36^] Following this procedure, the complexation behavior of gramibactin toward iron(III) in aqueous solution was studied by potentiometry using EDTA as competing ligand. Along this line, several measurements were performed at various molar ratios between gramibactin, EDTA, and iron(III). The corresponding evaluation of the experimental data was based on the EDTA protonation and EDTA‐iron(III) complex formation constants taken from literature and summarized in Table S2.[^32^, ^33^, ^34^, ^36^]

**Figure 3:**
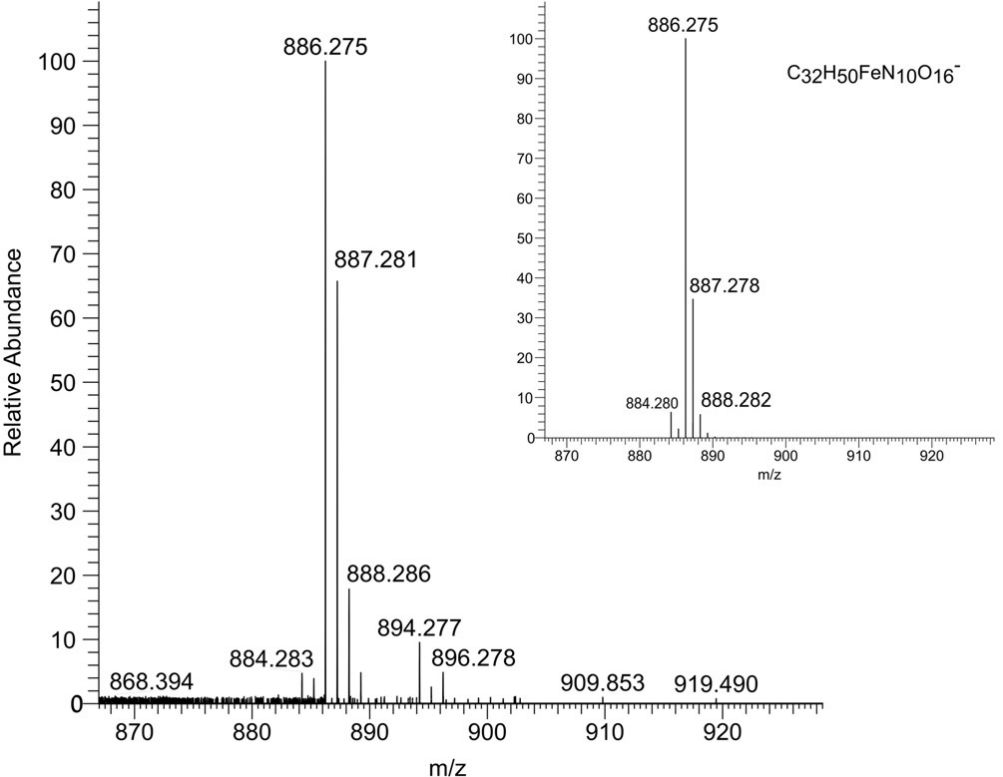
Experimental and theoretical^[39]^ (inset) high‐resolution LC‐MS spectra of the [M]^−^ species (C_32_H_50_FeN_10_O_16_^−^, *m*/*z=*886.275).

**Figure 4:**
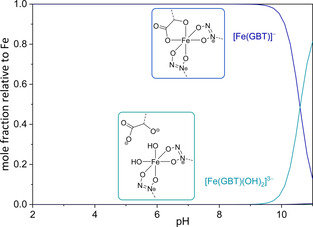
Distribution diagram of [Fe_p_(GBT)_q_H_r_] species as a function of pH in the Fe^3+^/GBT system in KCl(aq) at *I*=0.1 mol dm^−3^ and at *T=*298.15 K. *c*_GBT_=*c*_Fe3+_=1 mmol dm^−3^.

The analysis of a full set of potentiometric data obtained on the basis of the abovementioned aspects clearly evidenced the formation of two [Fe(GBT)H_r_] species within the investigated pH range (2≤pH≤11), namely [Fe(GBT)]^−^ and [Fe(GBT)(OH)_2_]^3−^, whose stability constants are reported in Table 2. Although surprising at first glance, the lack of a detectable stepwise deprotonation, i.e., the detection of a [Fe(GBT)(OH)]^2−^ species, is not unexpected, as such multistep protonation processes are not uncommon for cases involving strong multifunctional ligands and/or highly hydrolysable cations.[^37^, ^38^] The presence of the [Fe(GBT)]^−^ adduct was observed in high‐resolution LC‐MS as a peak with *m*/*z* at 886.275, corresponding to the chemical formula of [Fe(GBT)]^−^ (calc. for C_32_H_50_FeN_10_O_16_ 886.275; Figure 3), in the chromatogram of the supernatant of bacterial cultures. For a better visualization of the pH dependent behavior of the iron(III)‐gramibactin system, a speciation diagram is depicted in Figure 4.

**Table 2:** Iron(IIII) complex formation constants with gramibactin (GBT) in KCl_(aq)_ at *I*=0.1 mol dm^−3^ and *T=*298.15 K.

Species	*p*:*q*:*r*	log *β*_pqr_^[a]^
[Fe(GBT)]^−^	1:1:0	27.61±0.03 (27.56±0.09) ^[b]^
[Fe(GBT)(OH)_2_]^3−^	1:1:−2	6.42±0.06 (6.49±0.09) ^[b]^

[a] log*β*_pqr_ refer to the equilibrium: *p* Fe+*q* L+r H⇄[Fe_*p*_L_*q*_H_*r*_] ±95 % confidence interval; [b] values obtained from the fitting of the spectrophotometric measurements.

The [Fe(GBT)]^−^ species is the only iron species present in the pH range from 2.0 to ca. 9.0. Above this pH value, the formation of the corresponding dihydroxido species [Fe(GBT)(OH)_2_]^3−^ occurs. In any case, all iron(III) is fully complexed by gramibactin within the investigated pH range. Worth mentioning is also the fact that the inclusion of the polynuclear species Fe_12_(OH)_34_ in the model did not affect the results. Indeed, taking this species into account in the calculations, we obtained log *β*_110_=27.64±0.05 and log *β*_11‐2_=6.37±0.09 for [Fe(GBT)]^−^ and [Fe(GBT)(OH)_2_]^3−^, respectively, which is, within the experimental error, coincident with the values obtained not considering Fe_12_(OH)_34_ in the model (see Table 2).

**Figure 5:**
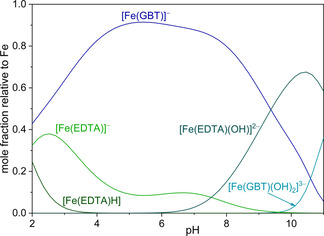
Distribution diagram of [Fe_p_L_q_H_r_] species as a function of pH value in the Fe^3+^/EDTA/GBT system in KCl_(aq)_ at *I*=0.1 mol dm^−3^ and at *T=*298.15 K. *c*_GBT_=*c*_EDTA_=*c*_Fe3+_=1 mmol dm^−3^.

The suitability of EDTA as competing ligand with gramibactin toward iron(III) complexation is demonstrated by the speciation diagram depicted in Figure 5 for the iron(III)/EDTA/gramibactin system considering a 1:1:1 ratio and an iron(III) concentration of *c*_Fe_=1 mmol dm^−3^. Under these experimental conditions and at a pH≈2, ca. 60 % of the iron(III) is coordinated by EDTA, while only ca. 40 % is coordinated by gramibactin. This allows to monitor the formation of the [Fe(GBT)]^−^ species, that reaches the maximum concentration at pH≈5.0, and consequently gives the possibility to accurately determine its formation constant using the ligand‐competition approach. Moreover, the speciation diagram in Figure 5 can also be useful to assess the distribution of iron(III) under conditions that are frequently observed in bacterial growth media, where EDTA is used as chelating agent to keep iron in solution.^[40]^

**Figure 6:**
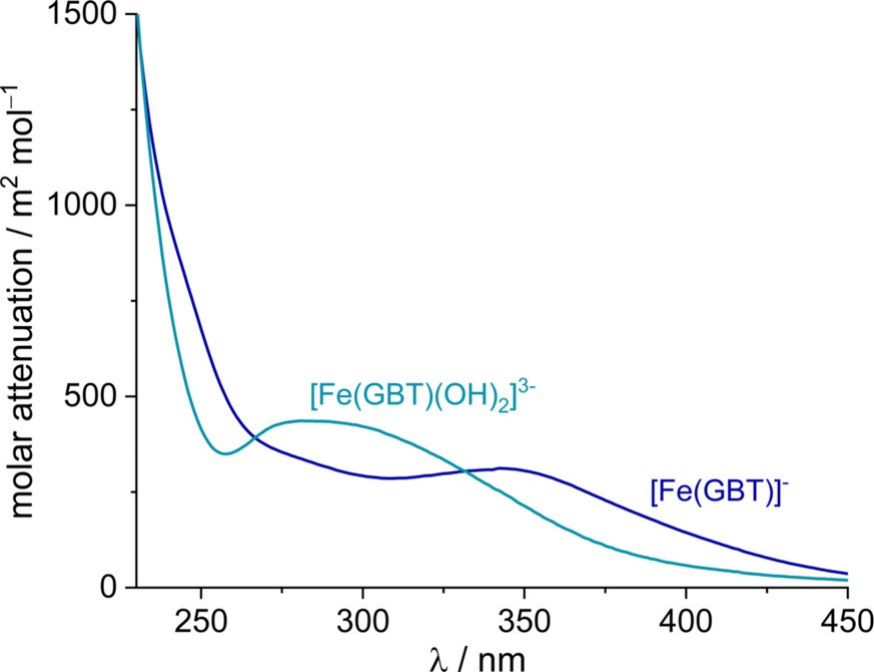
Calculated molar absorbance spectra for the species [Fe(GBT)] ^−^ and [Fe(GBT)(OH)_2_]^3−^.

The results obtained by potentiometric measurements were confirmed by UV/Vis spectrophotometry (see Figure S1). The analysis of the experimental data obtained by spectrophotometric titrations using the HypSpec program^[27]^ for fitting allowed for the determination of the stability constants of the iron‐gramibactin species [Fe(GBT)]^−^ and [Fe(GBT)(OH)_2_]^3−^ as log *β*_110_=27.56±0.09 and log *β*_11‐2_=6.49±0.09, respectively, which is in perfect agreement with the values obtained by potentiometry (see Table 2). The calculated spectra depicted in Figure 6 reveal that the [Fe(GBT)]^−^ species is characterized by a band centered at 343 nm (*ϵ*≅312 m^2^ mol^−1^) and a shoulder at *λ*≅240 nm, while for the dihydroxido species [Fe(GBT)(OH)_2_]^3−^ the observed absorption band appears at ca. 280 nm (*ϵ*≅436 m^2^ mol^−1^). A list of the calculated molar absorbance as a function of the wavelength of all gramibactin species present under these conditions is summarized in Table S3.

For the [Fe(GBT)]^−^ species, metal binding is clearly evidenced by a hypsochromic shift of the ligand absorption band upon coordination (from 246 to 240 nm), which is associated with a dramatic decrease in intensity. This is in agreement with what is reported in literature, namely for the iron(III) complex of the *N*‐nitroso‐*N*‐methyl‐hydroxylamine ligand (*λ*_max_: [L]^−^=246 nm, [FeL_3_]=242 nm).[^19^, ^30^] Moreover, upon coordination of the gramibactin ligand, an additional band for the [Fe(GBT)]^−^ species is observed at 343 nm (Figure S2), which can be assigned to a ligand‐to‐metal charge transfer between the α‐hydroxocarboxylate hydroxy oxygen donor and iron(III) ion.^[24]^ In the case of the dihydroxido species [Fe(GBT)(OH)_2_]^3−^, the latter ligand‐to‐metal charge transfer band disappears and a new band is found at around 280 nm.

Based on these observations, it is tempting to attribute the observed spectral changes between the two iron(III) gramibactin species, i.e., [Fe(GBT)]^−^ and [Fe(GBT)(OH)_2_]^3−^, to the loss of the α‐hydroxocarboxylate moiety from the ferric center, for which the octahedral coordination sphere is completed by two hydroxido ligands.

### Sequestering ability of gramibactin and its pH dependence

As emphasized before, a general interest of this study is to provide a comparative assessment of the sequestering ability of gramibactin as a representative example of the new class of diazeniumdiolate siderophores based on its ability for the sequestration of iron. This is fundamental to understand the role of gramibactin in the soil microenvironment, particularly in view of the competitive presence of other chelators. Several compounds have been reported as siderophores for mobilizing and/or transporting iron, such as nicotianamine in higher plants.^[41]^ Other specific phytosiderophores (PS) are secreted from the roots of graminaceous monocotyledonous plants during iron deficiency, like mugineic acid (MA) and its derivative, deoxymugineic acid (DMA).^[42]^ Furthermore, a large number of microbial siderophores (MS), ranging from catecholate‐type (e.g., enterobactin, amonabactin T) to hydroxamate‐type (e.g., desferrioxamine B, E and desferriferrichrome), are also present in all soil environments.^[43]^

Therefore, it is important to evaluate and compare the sequestering ability of gramibactin and other siderophores toward iron. However, the simple comparison of the iron‐siderophore formation constants is in most cases not sufficient to address this question. Indeed, other factors need to be considered, such as differences in the denticity of the ligands, in their coordination modes, and in their acid‐base properties (protonation reactions are competitive with respect to the metal complex formation, since hydrogen ions compete for the same binding sites).[^44^, ^45^] With all this in mind, several parameters have been defined in order to compare the relative strength of different metal chelating agents.[^44^, ^46^, ^47^] For the classification of the sequestering ability of gramibactin we will make use of two specific parameters, namely the pM value (defined as pM=−log[M], where [M], in the particular case of iron(III), represents the concentration of the free aqueous ion [Fe(H_2_O)_6_]^3+^ when *c*_L_/*c*_M_=10, *c*_M_=10^−6^ mol dm^−3^, pH 7.4) originally introduced by Raymond particularly for the comparison of iron‐siderophore systems^[46]^ and the semiempirical parameter pL_0.5_ (representing the total ligand concentration required to sequester 50 % of the metal cation under the given conditions of the system), which is the result of efforts to establish a parameter that is easy to use and less susceptible to misinterpretations.^[44]^ Although the two parameters are defined by the concentration of different species in the relevant solution equilibria (i.e., metal ion in case of pM and ligand for pL_0.5_), for both cases a larger value indicates a stronger sequestering ability of the ligand under investigation.

**Figure 7:**
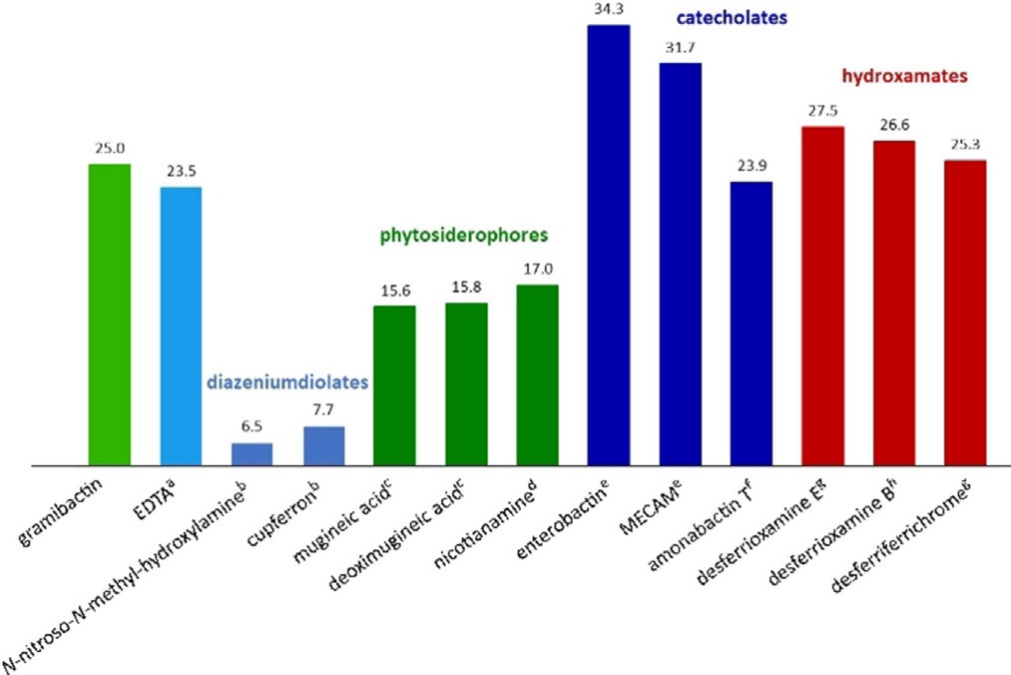
The pFe values of gramibactin and relevant ligands such as EDTA, diazeniumdiolates compounds, phytosiderophores, and microbial siderophores. The values are determined using the protonation and iron(III) complex formation constants reported in: [a] ref. [36], [b] ref. [31], [c] ref. [51], [d] ref. [5], [e] ref. [52], [f] ref. [53], [g] ref. [54], and [h] ref. [55].

Before we start to evaluate the particular numbers, a caveat has to be placed here. The generally perceived benefit of the pM value, which leads to its widespread use, is mainly related to its intuitive definition. However, this apparent advantage comes at the expense of frequent misuse of this parameter, particularly by neglecting Raymond's original rules and definition (*c*_L_/*c*_M_=10, *c*_M_=10^−6^ mol dm^−3^, pH 7.4).^[46]^ Therefore, we recalculated the pM values for all relevant chelators,[^1^, ^48^, ^49^, ^50^] even if they have been reported in literature for some cases, to avoid any comparison with practically inconsistent numbers. For a detailed and educational discussion on this topic see the Supporting Information and for example refs [44] and [47]. At first, we will focus on the pM value, as this is one of the most frequently applied metric parameters that is also used for other biologically and/or environmentally relevant metal ions such as gallium(III) and copper(II), although it was specifically introduced for the comparison of iron‐siderophore systems.^[46]^ In any case, a larger pM value corresponds to a lower concentration of the free metal ion in solution at equilibrium and, in principle, to a higher affinity of the relevant ligand for the metal ion studied. Consequently, the pFe value for the iron(III)‐gramibactin system has been calculated (*c*_L_/*c*_M_=10, *c*_M_=10^−6^ mol dm^−3^, pH 7.4) and is depicted in Figure 7 as a graphical comparison with the pFe values of different other classes of siderophores. The values recalculated in this work are based on the respective protonation and iron(III) complex formation constants taken from the original publications (see Table 3), using the iron(III) hydrolysis constants summarized in Table S2. This is particularly important in order to obtain a comparable set of pFe values, since such a comparison is only rigorously valid if all calculations have been performed following the same rules and applying the same conditions.

**Table 3:** Protonation and iron(III) complex formation constants of gramibactin, EDTA, selected diazeniumdiolates and siderophores, and corresponding pFe and pL_0.5_ values at pH 7.4.

	log *β*_pqr_^[a]^																pFe	pL_0.5_
	0:1:1	0:1:2	0:1:3	0:1:4	0:1:5	0:1:6	1:1:0	1:1:1	1:1:2	1:1:3	1:1:4	1:1:‐1	1:1:‐2	1:3:0	2:3:0	2:3:2		
Gramibactin^[b]^	10.9	16.7	21.5	27.8	–	–	27.6	–	–	–	–	–	6.4	–	–	–	25.0	15.5

DIAZENIUMDIOLATES																		
*N‐*nitroso‐*N*‐methyl‐ hydroxylamine^[c]^	–	–	–	–	–	–	–	–	–	–	–	–	–	15.7	–	–	6.5	5.2
Cupferron^[c]^	–	–	–	–	–	–	–	–	–	–	–	–	–	17.1	–	–	7.7	5.7

PHYTOSIDEROPHORES																		
Mugineic acid^[d]^	9.9	17.8	21.0	–	–	–	17.7	–	–	–	–	1.4	–	–	–	–	15.6	6.0
Deoximugineic acid^[d]^	10.0	18.3	21.4	–	–	–	18.4	–	–	–	–	2.2	–	–	–	–	15.8	6.3
Nicotianamine^[e]^	10.2	19.3	26.3	28.5	–	–	20.6	–	–	–	–	–	–	–	–	–	16.3	6.8

OTHER SIDEROPHORES																		
Enterobactin^[f]^	12.1	24.2	36.3	44.9	52.4	58.4	49.0	54.0	57.5	60.0	–	–	–	–	–	–	34.3	24.8
MECAM^[f]^	12.1	24.2	36.3	44.7	52.1	58.0	46.0	53.2	59.2	63.7	67.5	–	–	–	–	–	31.7	22.2
CYCAM^[g]^	12.1	24.2	36.3	45.6	54.2	62.1	40.0	49.0	56.6	62.5	–	–	–	–	–	–	25.2	15.6
Amonabactin T^[h]^	12.1	24.2	33.0	40.7	47.7	–	34.3	–	–	–	–	–	–	–	86.3	105.1	23.9	14.3
Desferrioxamine E^[i]^	9.9	19.3	28.0	–	–	–	32.4	32.4	–	–	–	–	–	–	–	–	27.5	18.0
Desferrioxamine B^[j]^	9.7	18.7	27.1	–	–	–	30.5	31.4	–	–	–	–	–	–	–	–	26.5	17.0
Desferriferrichrome A^[i]^	9.8	18.8	26.9	–	–	–	29.1	30.5	–	–	–	–	–	–	–	–	25.3	15.7
																		
EDTA^[k]^	10.2	16.4	19.1	21.1	–	–	25.1	27.0	–	–	–	17.6	–	–	–	–	23.5	13.9

[a] log *β*_pqr_ refer to the equilibrium: *p* Fe+*q* L+*r* H=Fe_*p*_L_*q*_H_***r***_, [Eq. (1)]. [b] This work; *I*=0.1 mol dm^−3^ (KCl) and *T=*298.15 K. [c] Ref. [31]; EtOH, *T* not indicated. [d] Ref. [51]; *I*=0.1 mol dm^−3^ (KNO_3_) and *T=*293.15 K. [e] Ref. [5]; *I*=0.1 mol dm^−3^ (KCl) and *T=*298.15 K. [f] Ref. [52]; *I*=0.1 mol dm^−3^ (KCl) and *T=*298.15 K. [g] Ref. [46]; *I*=0.1 mol dm^−3^ (KNO_3_) and *T=*298.15 K. [h] Ref. [53]; *I*=0.1 mol dm^−3^ (KCl) and *T=*298.15 K. [i] Ref. [54]; *I*=0.1 mol dm^−3^ (NaNO_3_) and *T=*293.15 K. [j] Ref. [55]; *I*=0.1 mol dm^−3^ (NaClO_4_) and *T=*293.15 K. [k] Ref. [36]; *I*=0.1 mol dm^−3^ (KNO_3_) and *T=*298.15 K.

The pFe value for gramibactin (25.0) is of the same order of magnitude as for known hydroxamate‐type siderophores such as desferrioxamine E (27.5), desferrioxamine B (26.5) and desferriferrichrome (25.3). However, enterobactin (34.3), a catecholate‐type siderophore, presents a much higher iron(III) chelating efficacy. Regarding the phytosiderophores mugineic acid, deoximugineic acid, or nicotianamine, the pFe value of gramibactin is 9 log units larger (25 vs. ≈16), while in comparison with some mono‐diazeniumdiolate ligands, such as *N*‐nitroso‐*N*‐methyl‐hydroxylamine or *N*‐nitroso‐*N*‐phenyl‐hydroxylamine (cupferron), the pFe value of gramibactin is even 18 log units larger. The observed effects reflect the differences in the coordinating groups as well as the number and type of donor atoms present in the ligand. The former is particularly evident, as both gramibactin and the phytosiderophores are hexadentate ligands. In fact, the diazeniumdiolate moieties present in gramibactin show a considerably better chelating affinity for iron(III) than the functional groups of the phytosiderophores (see Scheme S1). On the other hand, when comparing the chelating ability of ligands bearing the same chelating groups (*N*‐nitroso‐*N*‐hydroxylamine), it is observed that the denticity of the ligand is now the dominant effect. This is obvious from the comparison of gramibactin containing two diazeniumdiolate moieties, while in *N*‐nitroso‐*N*‐methyl‐hydroxylamine and *N*‐nitroso‐*N*‐phenylhydroxylamine (cupferron) only one chelating group is available to coordinate the metal ion. For the latter cases, 1:3 metal to ligand species are formed, whereas for gramibactin a 1:1 complex is found. Consequently, the difference in pFe value (25 vs. 7) can be roughly attributed to the lower stabilization of the bidentate ligand due to the loss of the chelate effect.[^54^, ^56^, ^57^] Finally, it is also worth comparing the pFe value of gramibactin with that of EDTA, as the latter is an integral part of most growth media used in laboratory studies, which shows that chelation by gramibactin is thermodynamically favored in terms of the pFe value by about 1.5 log units.

In order to overcome the not insignificant drawbacks of the pM value (see Supporting Information), the parameter pL_0.5_ has been introduced, which will be used in the following to compare the sequestering ability of gramibactin with the relevant types of siderophores already mentioned. In contrast to the pM value, the semiempirical parameter pL_0.5_ does not refer to the concentration of the free metal ion, but rather represents the total ligand concentration needed for sequestration of half of the metal cation (present as trace) under the given conditions of the system investigated.^[44]^ In the way the parameter pL_0.5_ was conceived and is being used in the calculations, it represents the effective sequestering ability of a ligand and can be used to make all kinds of possible comparisons (for further details see Supporting Information).

**Figure 8:**
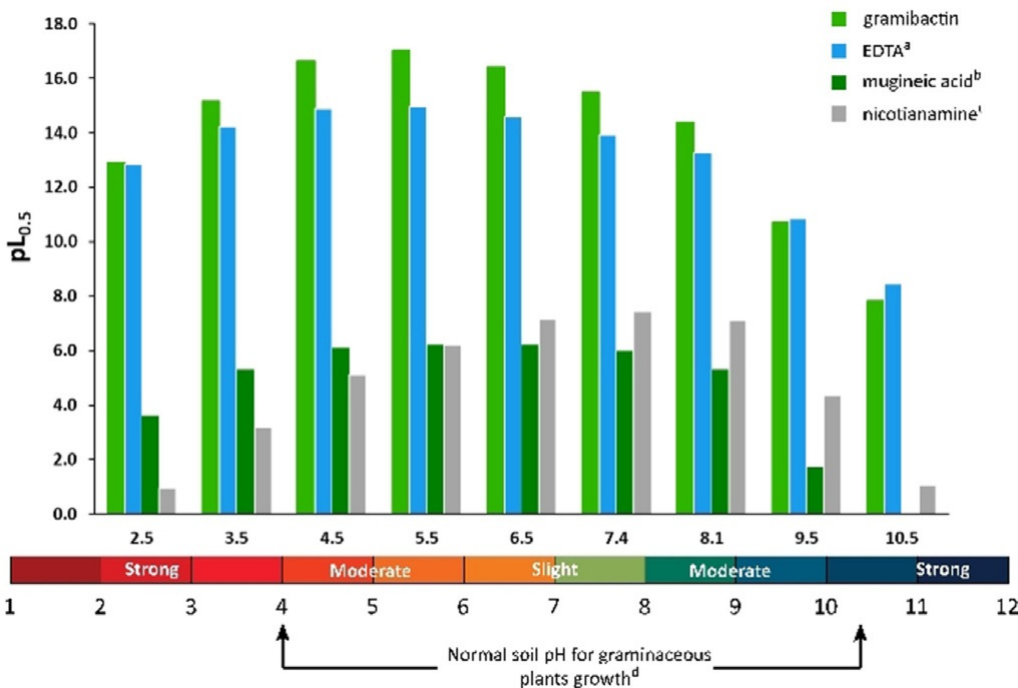
The pH profile of the pL_0.5_ values for gramibactin, EDTA and relevant phytosiderophores. Values determined using thermodynamic data reported in [a] ref. [44], [b] ref. [56], and [c] ref. [5]; [d] adapted from ref. [30].

The pL_0.5_ values for the iron(III) sequestration calculated for gramibactin and the other relevant ligands at pH 7.4 are summarized in Table 3. To address the situation in the soil microenvironment, the pH profiles of the pL_0.5_ values for gramibactin, EDTA and the two phytosiderophores mugineic acid and nicotianamine were also calculated and depicted in Figure 8 (cf. Table S4).

The obtained pL_0.5_ values show basically the same trends as observed for the pFe values (cf. Figure 7 and Table 3). Although the numbers of both parameters cannot be directly compared, it is generally observed that the numbers calculated for the pL_0.5_ values are about 10 log units smaller than the corresponding pFe values, with the simple bidentate diazeniumiolate ligands being the only exception. This leads to the rather surprising effect that the pL_0.5_ values for the iron(III) sequestration calculated of these bidentate diazeniumiolate ligands are in the same order of magnitude as those of the examples considered here for the class of phytosiderophores (mugineic acid, deoximugineic acid, and nicotianamine).

The same basic trends are observed for the calculated pL_0.5_ values upon variation of the pH when gramibactin is compared with EDTA and the two phytosiderophores mugineic acid and nicotianamine (see Figure 8). For all pH values considered, gramibactin is a markedly stronger chelating agent for iron(III) than the investigated phytosiderophores. Interestingly, even EDTA is a stronger iron(III) chelator with respect to the phytosiderophores. Compared to EDTA, however, gramibactin exhibits a much higher sequestering ability at most of the pH values under consideration. For example, at a pH of 7.4, a pL_0.5_ value that is 1.6 log units larger (gramibactin: 15.5 vs. EDTA: 13.9) means that under the same experimental conditions a 40‐fold lower concentration of gramibactin is required for the chelation of 50 % of the iron(III) in solution. In general, the ability of gramibactin to mobilize iron(III) is higher in the pH range from 4.5 and 6.5 (pL_0.5_ values of 16.6 and 16.4, respectively) with a maximum pL_0.5_ value of 17.0 at pH 5.5 and significantly decreases down to a value of about 8 at very high pH values. In addition, the generally observed decrease of sequestering ability of all four chelators analyzed at high pH values is due to competition with hydroxide ions, i.e., the iron(III) hydrolysis. Nevertheless, both gramibactin and EDTA can compete in a more efficient way with the hydrolysis than the phytosiderophores, presenting a pL_0.5_ of about 11 at pH 9.5 vs. a pL_0.5_ of less than 2 for mugineic acid.

In fact, the pronounced efficiency of iron(III) chelation by gramibactin reinforces the hypothesis that numerous graminaceous plants can exploit this microbial siderophore as iron source. Moreover, sequestration with gramibactin stabilizes iron(III) and thereby prevents its reduction to iron(II), which is a common process occurring in soil with low pH due to the presence of high concentrations of anaerobic bacteria. This fact is important since it is known that iron(II) acts as an antagonistic element on the uptake of essential nutrients (e.g., phosphorous, potassium, and zinc) by plants.^[26]^

From all these observations, either considering the pL_0.5_ or pFe values, a ligand‐exchange reaction of gramibactin and, for instance, mugineic acid (MA), is not expected to occur. Indeed, the formation of the complex [Fe(MA)]^−^ can only be relevant for an extremely large excess of the mugineic acid (*c*_MA_/*c*_GBT_≥10^8^). Consequently, [Fe(GBT)]^−^ remains the major species in solution in the pH range from 4 to 6.5, even when competing with relevant phytosiderophores (Figure S3).

## Conclusions

The iron sequestration behavior of gramibactin, an archetype for the new class of diazeniumdiolate siderophores, has been investigated. Gramibactin is produced by *Paraburkholderia graminis* and contains two *N*‐nitroso‐*N*‐hydroxylamine (diazeniumdiolate) chelating groups as well as one α‐hydroxocarboxylate. Diazeniumdiolates are a particularly interesting class of compounds due to their reported pharmacological potential, being only recently identified as siderophores, which together creates additional interest in the speciation and sequestration properties of gramibactin.

Toward this end, the acid‐base properties of gramibactin have been investigated by potentiometric measurements and can be described by four protonation steps, where the relevant log *K*_1r_ values (cf. Table 1) correspond to one hydroxyl (10.94), two *N*‐nitroso‐*N*‐hydroxylamine (5.71 and 4.87), and the carboxylate group (2.27). Gramibactin forms highly stable complexes with iron(III) ions over a wide range of pH values. The stability constants of the formed ferric gramibactin species were determined by potentiometric and spectrophotometric methods using the ligand competition approach with EDTA as competing ligand. [Fe(GBT)]^−^ is the only species present in the pH range from 2 to about 9, while at higher pH values the formation of the iron‐gramibactin dihydroxido species [Fe(GBT)(OH)_2_]^3−^ is observed.

The sequestering ability of gramibactin toward iron(III) ions was evaluated by means of metric parameters such as pFe and pL_0.5_. In terms of pFe, we could observe that at pH 7.4 gramibactin shows a much higher sequestering ability than EDTA (25.0 vs. 23.5), anticipating that, in the presence of EDTA as competitor ligand, the thermodynamic equilibrium is shifted toward gramibactin by a factor of 1.5 log units. The same result is obtained when the pL_0.5_ parameters are compared at the same pH value, for which gramibactin has a pL_0.5_ value that is 1.6 log units higher than that of EDTA (15.5 vs. 13.9), corresponding to a 40‐fold lower concentration of gramibactin required for chelating 50 % of the iron(III) ions in solution, as compared to EDTA. Furthermore, the pFe value of 25.0 is of the same order of magnitude as observed for known hydroxamate‐type siderophores. Considering the entire investigated pH range, based on the pL_0.5_ parameter, from very acidic to highly basic conditions, a dominant sequestering ability is found for gramibactin when compared with the phytosiderophores mugineic acid and nicotianamine. In fact, the highest sequestering ability for gramibactin occurs at moderate acidic conditions, suggesting some activity of gramibactin as a siderophore produced by the bacteria in the rhizosphere of graminaceous plants to prevent the formation of iron(II), thereby increasing their tolerance toward acidic soils.

These findings directly point to the question of the complexing ability of gramibactin toward iron(II), which will be part of future experiments. This is expected to provide a better understanding of its role as siderophore and in reducing environments. In addition, future efforts will also include studies on the sequestration ability of gramibactin toward zinc(II) and copper(II) ions, aiming at a possible application of this outstanding ligand as antibiotic, antifungal or even antitumoral agent, either by inhibition of metalloenzymes or simple metal depletion.

## Experimental Section

### Chemicals

KCl, Na_2_EDTA, and FeCl_3_⋅6 H_2_O solutions were prepared by weighing the corresponding salts, while HCl and KOH solutions were obtained by diluting concentrated ampoules. HCl and KOH were standardized against sodium carbonate and potassium hydrogen phthalate, respectively, previously dried in an oven at *T=*383 K for at least 2 h. FeCl_3_ solutions were standardized against EDTA standard solutions.^[58]^ Gramibactin was isolated as described elsewhere.^[2]^ The purity of gramibactin was determined by HPLC and potentiometric titrations (≥99 %). All solutions were prepared using analytical grade water and grade A glassware. All chemicals were purchased from Sigma–Aldrich (and its brands) at the highest available purity.

### Apparatus and procedure for potentiometric measurements

Potentiometric titrations were carried out at *I*=0.1 mol dm^−3^ in KCl_(aq)_ and at *T=*298.15±0.1 K in thermostatted cells, using a Mettler Toledo DL50 apparatus, equipped with a Schott Instruments N6180 ISE‐H^+^ combined glass electrode. The estimated accuracy was ±0.20 mV and ±0.02 mL for potential and titrant volume readings, respectively. The apparatus was connected to a PC and automatic titrations were performed using the LabX light v1.05 software to control titrant delivery, data acquisition and to check for potential stability. All potentiometric titrations, including electrode calibrations in terms of free proton concentration (i.e., pH=−log [H^+^], not activity), were carried out as reported elsewhere.^[35]^ Here, the titrant solutions were prepared by addition of different amounts of gramibactin (7×10^−4^≤c_GBT_/mol dm^−3^≤1×10^−3^), EDTA and Fe^3+^ with different GBT:Fe:EDTA ratios, together with the supporting electrolyte (KCl) to obtain the pre‐established ionic strength value (*I*=0.1 mol dm^−3^). In all samples, known slight excess of strong acid (HCl) was added in the titrant solution, in order to lower the starting pH of measurements. All the measurements were performed by titrating 10 to 20 mL of the titrant solution with standard KOH_(aq)_ up to pH≈11. 80–100 points were collected for each titration.

### Apparatus and procedure for spectrophotometric measurements

A Shimadzu UV‐1800 UV/Vis spectrophotometer was used to perform the spectrophotometric titrations, carried out at *I*=0.1 mol dm^−3^ in KCl_(aq)_ and *T=*298.15±0.1 K in a glass cuvette (1 cm of path length) placed in the spectrometer equipped with a thermostatted cell holder. An inoLab pH 7110 equipped with a ScienceLine Type N6000A combined ISE‐H^+^ glass electrode (SI analytics) was used for pH readings, after its calibration (in terms of free concentration, pH≡−log[H^+^]) before each experiment.^[22]^ Measurements were performed by titrating 2.2 cm^3^ of the titrant solution with standard KOH_(aq)_ solutions up to pH ∼11. Titrant solutions consisted of different amounts of GBT (3×10^−5^≤c_GBT_/mol dm^−3^≤6×10^−5^) and Fe^3+^ (1.5×10^−5^≤c_Fe_/mol dm^−3^≤6×10^−5^), HCl excess (*c*_H_=5–8 mmol dm^−3^), together with the supporting electrolyte (KCl) in order to adjust the desired ionic strength (*I*=0.1 mol dm^−3^). The homogeneity of the solutions during the titration was maintained by magnetic stirring.

### Apparatus and procedure for high‐resolution LC‐MS measurements

High‐resolution LC‐MS measurements were carried out on a Thermo Fisher Scientific Exactive Orbitrap with an electrospray ion source using a Betasil 100‐3 C18 column (150×2.1 mm) and an elution gradient (solvent A: H_2_O+0.1 % HCOOH, solvent B: acetonitrile, gradient: 5 % B for 1 min, 5 % to 98 % B in 15 min, 98 % B for 3 min, flow rate: 0.2 mL min^−1^, injection volume: 5 μL).

### Calculations

The BSTAC4^[59]^ computer program was used for determination of all the parameters of the acid‐base potentiometric titrations as well as for determination of the complex formation constants. Spectrophotometric data were evaluated by HypSpec.^[27]^ For the generation of speciation and sequestration diagrams, the Hyss^[60]^ and ES4ECI^[59]^ programs were used. All complex formation constants are expressed considering the overall equilibrium according to Equation 2,

(2) $$p\;\mathrm{Fe}{}^{3+}+q\;L{}^{4-}+r\;H{}^{+}\vec{\leftarrow }\mathrm{Fe}{}_{p}L{}_{q}H{}_{r}{}^{\left(3p-4q+r\right)}$$

which is also valid for the ligand protonation (with *p*=0) or metal hydrolysis constants (when *q*=0 and *r*<0). Protonation constants are also expressed as stepwise equilibria according to Equation 3.

(3) $$H{}^{+}+\mathrm{LH}{}_{r-1}{}^{\left(r-5\right)}\vec{\leftarrow }\mathrm{LH}{}_{r}{}^{\left(r-4\right)}$$

The molar concentration scale (*c*, mol dm^−3^) is used to express formation constants, concentrations and ionic strength, while errors are expressed as ± standard deviation. For simplicity, and when not relevant, the charges of the various species are omitted. Ligand acronyms (GBT and EDTA) in species and equilibria refer to the fully deprotonated species (GBT)^4−^ and (EDTA)^4−^.

## Conflict of interest

The authors declare no conflict of interest.

## Supporting information

As a service to our authors and readers, this journal provides supporting information supplied by the authors. Such materials are peer reviewed and may be re‐organized for online delivery, but are not copy‐edited or typeset. Technical support issues arising from supporting information (other than missing files) should be addressed to the authors.

Supplementary
