# Supplementary material for: Iron Coordination Properties of Gramibactin as Model for the New Class of Diazeniumdiolate Based Siderophores
Source: Chemistry. 2021 Jan 14;27(8):2724–33. doi: 10.1002/chem.202003842 (PMC7898861; doi:10.1002/chem.202003842)
Supplement: Supplementary file 1 — Supplementary [file CHEM-27-2724-s001.pdf]

Chemistry–A European Journal

Supporting Information

Iron Coordination Properties of Gramibactin as Model for the New Class of Diazeniumdiolate Based Siderophores

Sofia Gama,*[a, b] Ron Hermenau,[c] Mariachiara Frontauria,[a] Demetrio Milea,[d] Silvio Sammartano,[d] Christian Hertweck,[c, e] and Winfried Plass*[a]

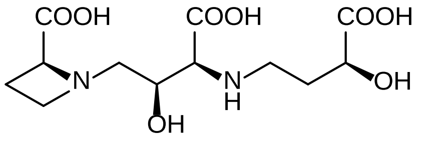

mugineic acid

(MA)

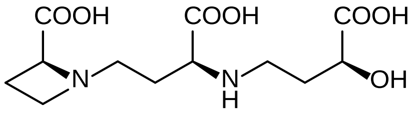

deoxymugineic acid

(DMA)

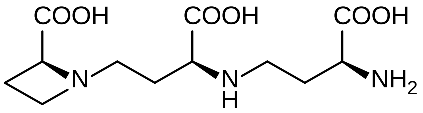

nicotianamine

(NA)

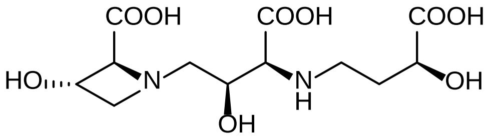

epi-hydroxymugineic acid (eHMA)

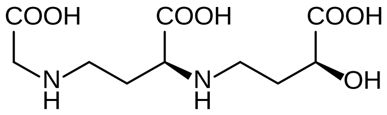

phytosiderophore I

(PSI)

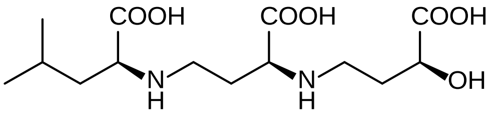

phytosiderophore II

(PSII)

Scheme S1. Phytosiderophores.

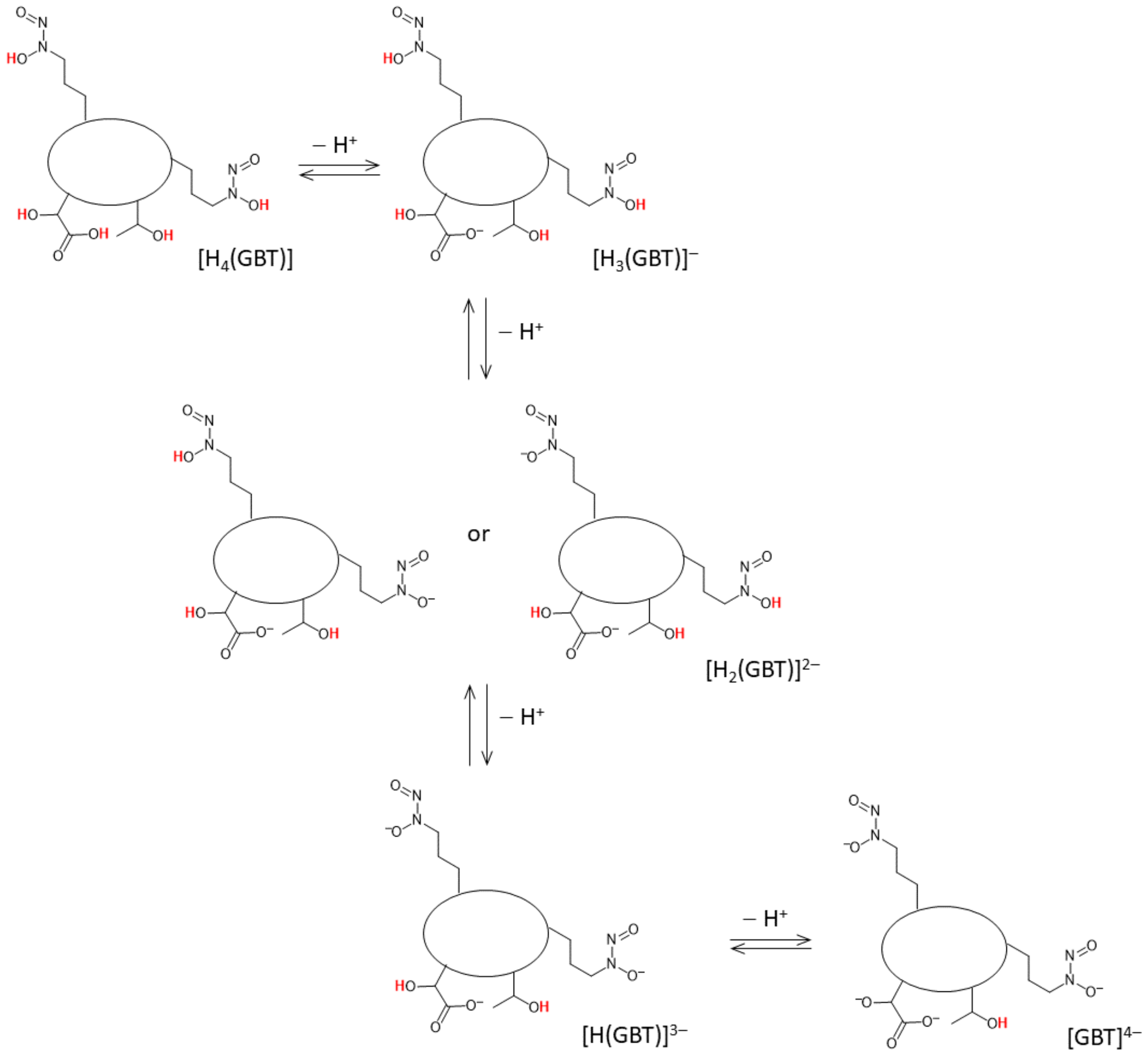

Scheme S2. Protonation sequence for gramibactin.

Table S1. Examples of *N*-nitroso-*N*-hydroxylamine compounds.

	Name	Structure	Produced by
**Natural compounds**	Alanosine (1966)[1]	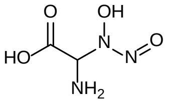	*Streptomyces alanosinicus*
	Fragin (1967)[2-3]	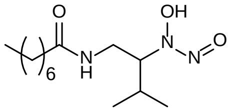	*Pseudomonas fragi*
	Dopastin (1974)[4]	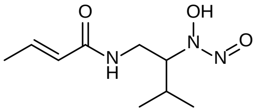	Pseudomonas No. BAC-125
	Nitrosofungin (1983)[5]	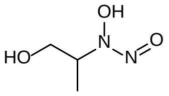	*Alcaligenes (UC 9152)* *Streptomyces plicatus UC 8272*
	Nitrosoxacins (1993)[6]	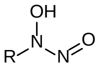 A, R = $(CH_3)_2CH(CH_2)_{13}-$ B, R = $CH_3(CH_2)_{15}-$ C, R = $(CH_3)_2CH(CH_2)_{11}-$	*Streptomyces*
	Valdiazen (2018)[7]	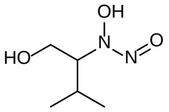	*Burkholderia cenocepacia*
**Synthetic compounds**	*N*-nitroso-*N*-phenylhydroxylamine (cupferron)[8]	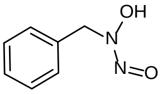	--
	N-nitroso-*N*-methyl-hydroxylamine[8]	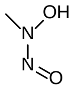	--

Table S2. Protonation constants for EDTA, Fe^{3+} hydrolysis and Fe^{3+}/EDTA complex formation constants (log β) [a], in $KCl_{(aq)}$ at I = 0.1 mol dm^{-3} and T = 298.15 K.

	Species	p:q:r	Log β [a]
**EDTA[b]**	$[HEDTA]^{3-}$	0:1:1	10.22
	$[H_2EDTA]^{2-}$	0:1:2	16.38
	$[H_3EDTA]^{-}$	0:1:3	19.09
	$[H_4EDTA]$	0:1:4	21.09
**Fe^{3+} [c]**	$[Fe(OH)]^{2+}$	1:0:-1	-2.17
	$[Fe(OH)_2]^{+}$	1:0:-2	-6.35
	$[Fe(OH)_3]$	1:0:-3	-14.25
	$[Fe(OH)_4]^{-}$	1:0:-4	-22.59
	$[Fe_2(OH)_2]^{4+}$	2:0:-2	-2.84
	$[Fe_3(OH)_4]^{5+}$	3:0:-4	-6.05
	$[Fe_{12}(OH)_{34}]^{2+}$	12:0:34	-55.2
**Fe^{3+}/EDTA[b]**	$[Fe(EDTA)]^{-}$	1:1:0	25.10
	$[Fe(HEDTA)]$	1:1:1	26.98
	$[Fe(EDTA)(OH)]^{2-}$	1:1:-1	17.57

[a] log β_{pqr} refers to the equilibrium: p Fe + q L + r H^+ = $Fe_pL_qH_r$

[b] Adapted from ref. [9]

[c] Adapted from refs. [10-12]

Table S3. Calculated molar absorbance spectra for the individual species of GBT and Fe^{3+}/GBT system.

λ / nm	$(GBT)^{4-}$	$[H(GBT)]^{3-}$	$[H_2(GBT)]^{2-}$	$[H_3(GBT)]^{-}$	$[H_4(GBT)]$	$[Fe(GBT)]^{-}$	$[Fe(GBT)(OH)_2]^{3-}$
205	8.74E+04	2.23E+04	2.26E+04	2.55E+04	1.99E+04	5.77E+04	8.77E+04
206	7.53E+04	2.03E+04	2.09E+04	2.35E+04	1.84E+04	5.71E+04	8.48E+04
207	6.38E+04	1.86E+04	1.94E+04	2.17E+04	1.70E+04	5.64E+04	8.03E+04
208	5.37E+04	1.72E+04	1.82E+04	2.03E+04	1.59E+04	5.49E+04	7.61E+04
209	4.55E+04	1.59E+04	1.72E+04	1.91E+04	1.51E+04	5.35E+04	7.15E+04
210	3.87E+04	1.49E+04	1.65E+04	1.82E+04	1.45E+04	5.18E+04	6.72E+04
211	3.35E+04	1.40E+04	1.61E+04	1.75E+04	1.42E+04	5.00E+04	6.34E+04
212	2.92E+04	1.32E+04	1.58E+04	1.70E+04	1.40E+04	4.81E+04	5.96E+04
213	2.58E+04	1.25E+04	1.56E+04	1.66E+04	1.40E+04	4.60E+04	5.58E+04
214	2.30E+04	1.19E+04	1.56E+04	1.64E+04	1.41E+04	4.37E+04	5.22E+04
215	2.08E+04	1.14E+04	1.57E+04	1.62E+04	1.43E+04	4.15E+04	4.87E+04
216	1.91E+04	1.10E+04	1.59E+04	1.62E+04	1.45E+04	3.92E+04	4.55E+04
217	1.78E+04	1.07E+04	1.61E+04	1.62E+04	1.48E+04	3.69E+04	4.23E+04
218	1.67E+04	1.05E+04	1.64E+04	1.63E+04	1.52E+04	3.46E+04	3.93E+04
219	1.59E+04	1.03E+04	1.66E+04	1.64E+04	1.55E+04	3.24E+04	3.65E+04
220	1.53E+04	1.02E+04	1.69E+04	1.65E+04	1.58E+04	3.02E+04	3.38E+04
221	1.48E+04	1.02E+04	1.72E+04	1.65E+04	1.61E+04	2.82E+04	3.13E+04
222	1.44E+04	1.02E+04	1.75E+04	1.66E+04	1.63E+04	2.63E+04	2.89E+04
223	1.41E+04	1.02E+04	1.78E+04	1.66E+04	1.65E+04	2.45E+04	2.67E+04
224	1.40E+04	1.04E+04	1.80E+04	1.65E+04	1.66E+04	2.27E+04	2.46E+04
225	1.39E+04	1.05E+04	1.82E+04	1.64E+04	1.66E+04	2.12E+04	2.28E+04
226	1.40E+04	1.08E+04	1.83E+04	1.63E+04	1.65E+04	1.97E+04	2.10E+04
227	1.42E+04	1.11E+04	1.84E+04	1.60E+04	1.63E+04	1.84E+04	1.94E+04
228	1.44E+04	1.14E+04	1.84E+04	1.57E+04	1.60E+04	1.72E+04	1.79E+04
229	1.47E+04	1.18E+04	1.83E+04	1.53E+04	1.56E+04	1.61E+04	1.66E+04
230	1.50E+04	1.23E+04	1.82E+04	1.49E+04	1.52E+04	1.51E+04	1.54E+04
231	1.55E+04	1.28E+04	1.80E+04	1.43E+04	1.46E+04	1.43E+04	1.43E+04
232	1.60E+04	1.33E+04	1.78E+04	1.37E+04	1.40E+04	1.35E+04	1.33E+04
233	1.65E+04	1.39E+04	1.75E+04	1.31E+04	1.33E+04	1.28E+04	1.24E+04
234	1.71E+04	1.45E+04	1.71E+04	1.24E+04	1.25E+04	1.22E+04	1.15E+04
235	1.76E+04	1.51E+04	1.68E+04	1.17E+04	1.17E+04	1.16E+04	1.07E+04
236	1.83E+04	1.57E+04	1.63E+04	1.09E+04	1.09E+04	1.12E+04	1.00E+04
237	1.89E+04	1.63E+04	1.59E+04	1.01E+04	1.00E+04	1.07E+04	9.35E+03
238	1.95E+04	1.70E+04	1.54E+04	9.24E+03	9.09E+03	1.03E+04	8.70E+03
239	2.01E+04	1.76E+04	1.49E+04	8.40E+03	8.17E+03	9.93E+03	8.10E+03
240	2.07E+04	1.82E+04	1.44E+04	7.60E+03	7.30E+03	9.60E+03	7.57E+03
241	2.12E+04	1.87E+04	1.39E+04	6.83E+03	6.48E+03	9.29E+03	7.08E+03
242	2.17E+04	1.92E+04	1.34E+04	6.08E+03	5.69E+03	8.99E+03	6.61E+03
243	2.20E+04	1.96E+04	1.29E+04	5.39E+03	4.95E+03	8.71E+03	6.20E+03
244	2.23E+04	2.00E+04	1.25E+04	4.75E+03	4.29E+03	8.43E+03	5.81E+03
245	2.25E+04	2.02E+04	1.20E+04	4.15E+03	3.68E+03	8.15E+03	5.45E+03
246	2.26E+04	2.04E+04	1.16E+04	3.60E+03	3.12E+03	7.86E+03	5.12E+03
247	2.26E+04	2.04E+04	1.12E+04	3.14E+03	2.66E+03	7.59E+03	4.83E+03
248	2.25E+04	2.04E+04	1.08E+04	2.73E+03	2.26E+03	7.31E+03	4.57E+03
249	2.22E+04	2.02E+04	1.04E+04	2.38E+03	1.93E+03	7.04E+03	4.35E+03
250	2.19E+04	1.99E+04	9.94E+03	2.07E+03	1.64E+03	6.76E+03	4.14E+03
251	2.14E+04	1.95E+04	9.55E+03	1.82E+03	1.41E+03	6.49E+03	3.98E+03
252	2.08E+04	1.90E+04	9.14E+03	1.61E+03	1.22E+03	6.23E+03	3.83E+03
253	2.01E+04	1.84E+04	8.72E+03	1.44E+03	1.06E+03	5.98E+03	3.71E+03
254	1.94E+04	1.78E+04	8.31E+03	1.29E+03	9.41E+02	5.74E+03	3.62E+03
255	1.85E+04	1.70E+04	7.87E+03	1.17E+03	8.17E+02	5.52E+03	3.56E+03
256	1.76E+04	1.62E+04	7.43E+03	1.06E+03	7.15E+02	5.30E+03	3.52E+03
257	1.66E+04	1.53E+04	6.98E+03	9.80E+02	6.39E+02	5.11E+03	3.50E+03
258	1.55E+04	1.43E+04	6.50E+03	8.99E+02	5.61E+02	4.92E+03	3.50E+03
259	1.45E+04	1.34E+04	6.03E+03	8.29E+02	4.95E+02	4.76E+03	3.51E+03
260	1.34E+04	1.23E+04	5.55E+03	7.75E+02	4.31E+02	4.60E+03	3.54E+03

261	1.23E+04	1.13E+04	5.09E+03	7.27E+02	3.73E+02	4.47E+03	3.58E+03
262	1.12E+04	1.04E+04	4.64E+03	6.87E+02	3.18E+02	4.35E+03	3.63E+03
263	1.02E+04	9.38E+03	4.19E+03	6.52E+02	2.68E+02	4.23E+03	3.68E+03
264	9.18E+03	8.43E+03	3.76E+03	6.18E+02	2.22E+02	4.13E+03	3.74E+03
265	8.22E+03	7.52E+03	3.36E+03	5.90E+02	1.82E+02	4.04E+03	3.80E+03
266	7.31E+03	6.66E+03	2.98E+03	5.67E+02	1.37E+02	3.97E+03	3.88E+03
267	6.47E+03	5.86E+03	2.63E+03	5.49E+02	1.05E+02	3.90E+03	3.94E+03
268	5.69E+03	5.11E+03	2.29E+03	5.33E+02	7.62E+01	3.84E+03	4.01E+03
269	5.01E+03	4.46E+03	2.01E+03	5.19E+02	5.05E+01	3.78E+03	4.06E+03
270	4.43E+03	3.88E+03	1.75E+03	5.10E+02	3.15E+01	3.74E+03	4.12E+03
271	3.89E+03	3.37E+03	1.52E+03	4.99E+02	-5.37E+00	3.69E+03	4.17E+03
272	3.45E+03	2.92E+03	1.31E+03	4.88E+02	-2.31E+01	3.65E+03	4.21E+03
273	3.09E+03	2.57E+03	1.15E+03	4.85E+02	-2.96E+01	3.61E+03	4.25E+03
274	2.76E+03	2.24E+03	9.94E+02	4.78E+02	-4.52E+01	3.58E+03	4.27E+03
275	2.50E+03	1.99E+03	8.75E+02	4.72E+02	-5.39E+01	3.54E+03	4.30E+03
276	2.30E+03	1.78E+03	7.76E+02	4.67E+02	-6.72E+01	3.51E+03	4.32E+03
277	2.13E+03	1.60E+03	6.84E+02	4.61E+02	-6.78E+01	3.48E+03	4.33E+03
278	1.99E+03	1.45E+03	6.12E+02	4.56E+02	-7.06E+01	3.45E+03	4.34E+03
279	1.88E+03	1.34E+03	5.50E+02	4.55E+02	-7.11E+01	3.43E+03	4.35E+03
280	1.80E+03	1.26E+03	5.03E+02	4.48E+02	-7.50E+01	3.40E+03	4.36E+03
281	1.73E+03	1.18E+03	4.59E+02	4.45E+02	-6.94E+01	3.37E+03	4.36E+03
282	1.67E+03	1.12E+03	4.19E+02	4.42E+02	-7.58E+01	3.34E+03	4.36E+03
283	1.63E+03	1.06E+03	3.89E+02	4.33E+02	-7.65E+01	3.31E+03	4.36E+03
284	1.58E+03	1.02E+03	3.65E+02	4.28E+02	-8.62E+01	3.28E+03	4.36E+03
285	1.55E+03	9.85E+02	3.39E+02	4.25E+02	-7.91E+01	3.26E+03	4.36E+03
286	1.51E+03	9.52E+02	3.21E+02	4.17E+02	-7.39E+01	3.23E+03	4.35E+03
287	1.49E+03	9.23E+02	3.03E+02	4.14E+02	-7.85E+01	3.20E+03	4.36E+03
288	1.47E+03	8.99E+02	2.89E+02	4.11E+02	-7.27E+01	3.17E+03	4.35E+03
289	1.45E+03	8.74E+02	2.71E+02	4.10E+02	-6.22E+01	3.15E+03	4.35E+03
290	1.43E+03	8.51E+02	2.58E+02	4.06E+02	-6.12E+01	3.13E+03	4.34E+03
291	1.40E+03	8.22E+02	2.42E+02	4.00E+02	-6.50E+01	3.10E+03	4.33E+03
292	1.36E+03	7.88E+02	2.17E+02	3.95E+02	-6.95E+01	3.07E+03	4.32E+03
293	1.33E+03	7.65E+02	2.07E+02	3.92E+02	-6.78E+01	3.05E+03	4.31E+03
294	1.31E+03	7.45E+02	1.98E+02	3.92E+02	-6.76E+01	3.03E+03	4.30E+03
295	1.27E+03	7.18E+02	1.87E+02	3.82E+02	-6.88E+01	3.01E+03	4.29E+03
296	1.24E+03	6.96E+02	1.77E+02	3.79E+02	-6.83E+01	2.98E+03	4.28E+03
297	1.21E+03	6.70E+02	1.67E+02	3.77E+02	-7.16E+01	2.97E+03	4.26E+03
298	1.18E+03	6.49E+02	1.61E+02	3.76E+02	-7.26E+01	2.95E+03	4.26E+03
299	1.16E+03	6.25E+02	1.50E+02	3.71E+02	-6.66E+01	2.94E+03	4.23E+03
300	1.12E+03	6.01E+02	1.41E+02	3.68E+02	-7.40E+01	2.92E+03	4.22E+03
301	1.09E+03	5.79E+02	1.32E+02	3.67E+02	-6.87E+01	2.91E+03	4.20E+03
302	1.06E+03	5.56E+02	1.23E+02	3.62E+02	-6.80E+01	2.90E+03	4.17E+03
303	1.04E+03	5.35E+02	1.09E+02	3.65E+02	-5.82E+01	2.89E+03	4.15E+03
304	1.00E+03	5.15E+02	1.07E+02	3.64E+02	-5.89E+01	2.88E+03	4.13E+03
305	9.78E+02	4.94E+02	9.67E+01	3.61E+02	-6.22E+01	2.87E+03	4.11E+03
306	9.22E+02	4.64E+02	8.55E+01	3.48E+02	-7.87E+01	2.86E+03	4.07E+03
307	8.98E+02	4.46E+02	7.97E+01	3.48E+02	-8.18E+01	2.86E+03	4.03E+03
308	8.75E+02	4.29E+02	7.56E+01	3.48E+02	-6.93E+01	2.86E+03	4.00E+03
309	8.37E+02	4.07E+02	6.30E+01	3.42E+02	-8.03E+01	2.85E+03	3.98E+03
310	8.20E+02	3.94E+02	6.03E+01	3.46E+02	-7.94E+01	2.86E+03	3.94E+03
311	8.00E+02	3.82E+02	5.62E+01	3.47E+02	-6.25E+01	2.86E+03	3.91E+03
312	7.72E+02	3.64E+02	5.64E+01	3.38E+02	-6.94E+01	2.87E+03	3.87E+03
313	7.47E+02	3.48E+02	4.76E+01	3.36E+02	-6.61E+01	2.87E+03	3.83E+03
314	7.27E+02	3.38E+02	3.76E+01	3.38E+02	-7.42E+01	2.88E+03	3.80E+03
315	7.12E+02	3.29E+02	4.31E+01	3.39E+02	-6.42E+01	2.88E+03	3.77E+03
316	6.91E+02	3.19E+02	3.74E+01	3.39E+02	-6.59E+01	2.89E+03	3.73E+03
317	6.71E+02	3.09E+02	3.40E+01	3.40E+02	-7.00E+01	2.90E+03	3.69E+03
318	6.55E+02	2.98E+02	2.88E+01	3.36E+02	-6.62E+01	2.91E+03	3.65E+03
319	6.40E+02	2.90E+02	1.94E+01	3.34E+02	-6.97E+01	2.92E+03	3.61E+03
320	6.42E+02	2.91E+02	2.46E+01	3.42E+02	-4.25E+01	2.93E+03	3.56E+03
321	6.18E+02	2.79E+02	2.31E+01	3.33E+02	-5.09E+01	2.93E+03	3.51E+03

322	5.93E+02	2.70E+02	1.30E+01	3.32E+02	-7.32E+01	2.95E+03	3.48E+03
323	5.88E+02	2.67E+02	1.53E+01	3.34E+02	-6.72E+01	2.96E+03	3.43E+03
324	5.68E+02	2.61E+02	9.62E+00	3.28E+02	-5.95E+01	2.97E+03	3.40E+03
325	5.62E+02	2.57E+02	4.94E+00	3.30E+02	-5.29E+01	2.98E+03	3.35E+03
326	5.48E+02	2.50E+02	-2.18E+00	3.25E+02	-6.45E+01	2.99E+03	3.30E+03
327	5.38E+02	2.53E+02	9.57E+00	3.28E+02	-6.69E+01	3.00E+03	3.25E+03
328	5.34E+02	2.47E+02	4.88E+00	3.29E+02	-5.97E+01	3.01E+03	3.21E+03
329	5.36E+02	2.48E+02	8.20E+00	3.29E+02	-3.99E+01	3.02E+03	3.15E+03
330	5.20E+02	2.48E+02	1.12E+01	3.27E+02	-4.80E+01	3.03E+03	3.11E+03
331	5.11E+02	2.44E+02	5.07E+00	3.25E+02	-4.92E+01	3.04E+03	3.07E+03
332	4.98E+02	2.45E+02	-1.78E+00	3.31E+02	-5.51E+01	3.05E+03	3.03E+03
333	4.96E+02	2.41E+02	1.54E+00	3.26E+02	-5.44E+01	3.05E+03	2.98E+03
334	4.75E+02	2.37E+02	4.56E+00	3.17E+02	-5.75E+01	3.06E+03	2.93E+03
335	4.80E+02	2.38E+02	2.78E+00	3.21E+02	-5.11E+01	3.06E+03	2.87E+03
336	4.70E+02	2.35E+02	9.40E-01	3.18E+02	-4.66E+01	3.07E+03	2.82E+03
337	4.62E+02	2.33E+02	-4.48E+00	3.18E+02	-5.17E+01	3.08E+03	2.77E+03
338	4.66E+02	2.37E+02	-5.82E+00	3.22E+02	-4.27E+01	3.08E+03	2.72E+03
339	4.46E+02	2.34E+02	8.01E+00	3.19E+02	-5.36E+01	3.09E+03	2.68E+03
340	3.88E+02	2.13E+02	-6.16E+00	2.86E+02	-9.98E+01	3.08E+03	2.62E+03
341	3.94E+02	2.12E+02	1.06E+00	2.83E+02	-7.40E+01	3.10E+03	2.57E+03
342	4.14E+02	2.26E+02	3.26E+00	3.06E+02	-5.55E+01	3.12E+03	2.54E+03
343	4.25E+02	2.33E+02	7.41E+00	3.12E+02	-4.71E+01	3.12E+03	2.50E+03
344	4.13E+02	2.28E+02	3.66E+00	3.06E+02	-3.90E+01	3.12E+03	2.44E+03
345	4.05E+02	2.27E+02	-2.60E+00	3.07E+02	-4.13E+01	3.11E+03	2.40E+03
346	3.89E+02	2.25E+02	3.79E-01	3.02E+02	-5.48E+01	3.10E+03	2.35E+03
347	3.81E+02	2.24E+02	1.88E+00	2.98E+02	-5.04E+01	3.10E+03	2.29E+03
348	3.74E+02	2.27E+02	1.07E+01	2.99E+02	-5.48E+01	3.09E+03	2.23E+03
349	3.67E+02	2.24E+02	7.76E+00	2.92E+02	-5.49E+01	3.07E+03	2.18E+03
350	3.68E+02	2.24E+02	8.29E+00	2.94E+02	-4.51E+01	3.06E+03	2.14E+03
351	3.70E+02	2.17E+02	-8.71E+00	2.98E+02	-4.83E+01	3.04E+03	2.09E+03
352	3.57E+02	2.19E+02	6.47E+00	2.93E+02	-5.35E+01	3.03E+03	2.04E+03
353	3.57E+02	2.17E+02	7.94E+00	2.86E+02	-3.89E+01	3.01E+03	1.99E+03
354	3.56E+02	2.20E+02	3.20E+00	2.90E+02	-3.80E+01	2.99E+03	1.94E+03
355	3.46E+02	2.14E+02	-9.94E-01	2.85E+02	-4.66E+01	2.96E+03	1.89E+03
356	3.43E+02	2.08E+02	-8.43E+00	2.82E+02	-4.70E+01	2.94E+03	1.83E+03
357	3.40E+02	2.10E+02	-1.78E+00	2.82E+02	-4.44E+01	2.91E+03	1.79E+03
358	3.40E+02	2.13E+02	-1.61E+00	2.85E+02	-3.96E+01	2.89E+03	1.75E+03
359	3.31E+02	2.11E+02	1.05E+01	2.81E+02	-3.86E+01	2.86E+03	1.71E+03
360	3.27E+02	2.07E+02	1.85E+00	2.78E+02	-4.28E+01	2.82E+03	1.66E+03
361	3.25E+02	2.10E+02	-2.32E+00	2.83E+02	-5.00E+01	2.79E+03	1.61E+03
362	3.26E+02	2.08E+02	-1.17E+00	2.78E+02	-3.79E+01	2.76E+03	1.57E+03
363	3.25E+02	2.08E+02	-1.73E+00	2.77E+02	-3.18E+01	2.73E+03	1.53E+03
364	3.27E+02	2.08E+02	-5.04E+00	2.80E+02	-2.51E+01	2.70E+03	1.50E+03
365	3.36E+02	2.12E+02	-2.36E+00	2.87E+02	-2.21E+01	2.66E+03	1.46E+03
366	3.07E+02	1.99E+02	-6.15E+00	2.69E+02	-3.98E+01	2.62E+03	1.42E+03
367	3.00E+02	1.97E+02	-5.99E+00	2.67E+02	-4.25E+01	2.58E+03	1.38E+03
368	3.00E+02	1.92E+02	-8.42E+00	2.61E+02	-4.74E+01	2.55E+03	1.33E+03
369	3.02E+02	1.91E+02	-1.01E+01	2.61E+02	-4.16E+01	2.51E+03	1.29E+03
370	3.06E+02	1.94E+02	-1.05E+01	2.67E+02	-3.62E+01	2.48E+03	1.27E+03
371	2.96E+02	1.86E+02	-1.46E+01	2.55E+02	-3.13E+01	2.43E+03	1.22E+03
372	2.94E+02	1.88E+02	-9.10E+00	2.56E+02	-4.61E+01	2.40E+03	1.19E+03
373	2.92E+02	1.85E+02	-1.54E+01	2.56E+02	-4.32E+01	2.36E+03	1.17E+03
374	2.85E+02	1.82E+02	-1.48E+01	2.52E+02	-4.25E+01	2.32E+03	1.14E+03
375	2.79E+02	1.77E+02	-1.35E+01	2.43E+02	-4.14E+01	2.28E+03	1.09E+03
376	2.88E+02	1.78E+02	-1.47E+01	2.47E+02	-3.91E+01	2.25E+03	1.07E+03
377	2.88E+02	1.79E+02	-1.26E+01	2.48E+02	-3.72E+01	2.21E+03	1.05E+03
378	2.78E+02	1.75E+02	-1.54E+01	2.41E+02	-4.70E+01	2.17E+03	1.01E+03
379	2.75E+02	1.71E+02	-1.89E+01	2.37E+02	-5.33E+01	2.14E+03	9.80E+02
380	2.76E+02	1.70E+02	-1.66E+01	2.36E+02	-4.88E+01	2.10E+03	9.53E+02
381	2.72E+02	1.72E+02	-1.16E+01	2.37E+02	-4.29E+01	2.07E+03	9.36E+02
382	2.76E+02	1.71E+02	-1.51E+01	2.37E+02	-4.08E+01	2.03E+03	9.17E+02

383	2.80E+02	1.69E+02	-1.26E+01	2.34E+02	-4.02E+01	2.00E+03	8.85E+02
384	2.73E+02	1.64E+02	-1.86E+01	2.29E+02	-4.03E+01	1.96E+03	8.62E+02
385	2.71E+02	1.61E+02	-1.83E+01	2.24E+02	-4.59E+01	1.93E+03	8.38E+02
386	2.68E+02	1.61E+02	-1.45E+01	2.23E+02	-4.35E+01	1.90E+03	8.20E+02
387	2.62E+02	1.57E+02	-1.60E+01	2.17E+02	-4.70E+01	1.86E+03	7.98E+02
388	2.63E+02	1.56E+02	-1.63E+01	2.17E+02	-4.50E+01	1.83E+03	7.84E+02
389	2.64E+02	1.54E+02	-1.59E+01	2.15E+02	-4.26E+01	1.80E+03	7.57E+02
390	2.64E+02	1.53E+02	-1.77E+01	2.15E+02	-4.11E+01	1.76E+03	7.45E+02
391	2.59E+02	1.51E+02	-1.56E+01	2.11E+02	-5.65E+01	1.73E+03	7.25E+02
392	2.64E+02	1.50E+02	-1.27E+01	2.09E+02	-4.39E+01	1.70E+03	7.01E+02
393	2.60E+02	1.46E+02	-1.68E+01	2.04E+02	-4.69E+01	1.66E+03	6.82E+02
394	2.63E+02	1.45E+02	-1.68E+01	2.03E+02	-4.18E+01	1.63E+03	6.64E+02
395	2.58E+02	1.44E+02	-1.56E+01	2.02E+02	-4.75E+01	1.60E+03	6.53E+02
396	2.56E+02	1.38E+02	-2.16E+01	1.97E+02	-4.56E+01	1.57E+03	6.39E+02
397	2.55E+02	1.37E+02	-1.92E+01	1.95E+02	-4.72E+01	1.53E+03	6.22E+02
398	2.56E+02	1.36E+02	-1.86E+01	1.94E+02	-4.95E+01	1.50E+03	6.06E+02
399	2.54E+02	1.33E+02	-1.92E+01	1.91E+02	-4.94E+01	1.47E+03	5.93E+02
400	2.55E+02	1.33E+02	-1.85E+01	1.91E+02	-5.01E+01	1.44E+03	5.83E+02
401	2.58E+02	1.34E+02	-1.72E+01	1.91E+02	-4.66E+01	1.41E+03	5.69E+02
402	2.58E+02	1.32E+02	-1.85E+01	1.89E+02	-4.49E+01	1.38E+03	5.53E+02
403	2.59E+02	1.30E+02	-2.07E+01	1.86E+02	-4.39E+01	1.35E+03	5.41E+02
404	2.60E+02	1.29E+02	-1.92E+01	1.85E+02	-4.32E+01	1.32E+03	5.29E+02
405	2.57E+02	1.25E+02	-2.18E+01	1.82E+02	-4.71E+01	1.30E+03	5.17E+02
406	2.52E+02	1.22E+02	-2.12E+01	1.78E+02	-4.82E+01	1.27E+03	5.08E+02
407	2.56E+02	1.24E+02	-1.96E+01	1.79E+02	-4.66E+01	1.24E+03	4.98E+02
408	2.56E+02	1.25E+02	-1.75E+01	1.80E+02	-4.73E+01	1.21E+03	4.90E+02
409	2.56E+02	1.22E+02	-1.96E+01	1.77E+02	-4.78E+01	1.19E+03	4.78E+02
410	2.55E+02	1.19E+02	-2.06E+01	1.73E+02	-4.46E+01	1.16E+03	4.66E+02
411	2.53E+02	1.18E+02	-2.11E+01	1.72E+02	-4.76E+01	1.13E+03	4.56E+02
412	2.55E+02	1.16E+02	-2.20E+01	1.71E+02	-4.35E+01	1.10E+03	4.47E+02
413	2.57E+02	1.18E+02	-2.17E+01	1.73E+02	-4.24E+01	1.08E+03	4.42E+02
414	2.54E+02	1.15E+02	-2.30E+01	1.70E+02	-4.57E+01	1.05E+03	4.28E+02
415	2.52E+02	1.11E+02	-2.38E+01	1.66E+02	-5.00E+01	1.02E+03	4.17E+02
416	2.70E+02	1.22E+02	-1.95E+01	1.80E+02	-3.40E+01	1.00E+03	4.18E+02
417	2.63E+02	1.16E+02	-1.97E+01	1.71E+02	-3.68E+01	9.72E+02	3.96E+02
418	2.55E+02	1.10E+02	-2.13E+01	1.63E+02	-4.28E+01	9.43E+02	3.82E+02
419	2.55E+02	1.08E+02	-2.24E+01	1.61E+02	-4.24E+01	9.19E+02	3.72E+02
420	2.52E+02	1.07E+02	-2.12E+01	1.59E+02	-4.39E+01	8.95E+02	3.66E+02
421	2.52E+02	1.05E+02	-2.25E+01	1.58E+02	-4.51E+01	8.70E+02	3.58E+02
422	2.54E+02	1.05E+02	-2.18E+01	1.59E+02	-4.52E+01	8.48E+02	3.52E+02
423	2.53E+02	1.04E+02	-2.18E+01	1.57E+02	-4.53E+01	8.26E+02	3.42E+02
424	2.53E+02	1.02E+02	-1.98E+01	1.54E+02	-4.40E+01	8.03E+02	3.33E+02
425	2.51E+02	1.01E+02	-2.15E+01	1.52E+02	-4.57E+01	7.80E+02	3.26E+02
426	2.51E+02	9.90E+01	-2.32E+01	1.52E+02	-4.73E+01	7.58E+02	3.19E+02
427	2.52E+02	9.88E+01	-2.35E+01	1.51E+02	-4.51E+01	7.37E+02	3.11E+02
428	2.50E+02	9.83E+01	-2.18E+01	1.51E+02	-4.70E+01	7.17E+02	3.07E+02
429	2.52E+02	9.80E+01	-2.34E+01	1.51E+02	-4.29E+01	6.96E+02	3.02E+02
430	2.50E+02	9.66E+01	-2.33E+01	1.50E+02	-4.60E+01	6.77E+02	2.98E+02
431	2.49E+02	9.51E+01	-2.40E+01	1.48E+02	-4.52E+01	6.57E+02	2.89E+02
432	2.52E+02	9.53E+01	-2.41E+01	1.49E+02	-4.49E+01	6.38E+02	2.82E+02
433	2.53E+02	9.51E+01	-2.37E+01	1.48E+02	-4.28E+01	6.21E+02	2.77E+02
434	2.49E+02	9.34E+01	-2.36E+01	1.46E+02	-4.37E+01	6.04E+02	2.73E+02
435	2.52E+02	9.35E+01	-2.47E+01	1.47E+02	-4.19E+01	5.85E+02	2.67E+02
436	2.53E+02	9.22E+01	-2.44E+01	1.46E+02	-4.22E+01	5.68E+02	2.60E+02
437	2.51E+02	9.04E+01	-2.52E+01	1.44E+02	-4.08E+01	5.52E+02	2.55E+02
438	2.51E+02	8.91E+01	-2.62E+01	1.43E+02	-4.15E+01	5.35E+02	2.50E+02
439	2.53E+02	9.05E+01	-2.53E+01	1.45E+02	-4.12E+01	5.20E+02	2.45E+02
440	2.55E+02	9.03E+01	-2.54E+01	1.45E+02	-4.01E+01	5.04E+02	2.39E+02
441	2.53E+02	8.83E+01	-2.59E+01	1.43E+02	-4.15E+01	4.88E+02	2.33E+02
442	2.52E+02	8.77E+01	-2.77E+01	1.43E+02	-4.20E+01	4.73E+02	2.33E+02
443	2.49E+02	8.57E+01	-2.71E+01	1.40E+02	-4.47E+01	4.57E+02	2.28E+02

444	2.49E+02	8.53E+01	-2.71E+01	1.40E+02	-4.36E+01	4.43E+02	2.22E+02
445	2.51E+02	8.58E+01	-2.64E+01	1.41E+02	-4.11E+01	4.31E+02	2.19E+02
446	2.52E+02	8.55E+01	-2.69E+01	1.40E+02	-3.81E+01	4.17E+02	2.14E+02
447	2.50E+02	8.40E+01	-2.83E+01	1.39E+02	-3.92E+01	4.04E+02	2.10E+02
448	2.49E+02	8.23E+01	-2.98E+01	1.37E+02	-3.81E+01	3.90E+02	2.05E+02
449	2.47E+02	8.19E+01	-2.86E+01	1.36E+02	-4.04E+01	3.77E+02	2.01E+02
450	2.48E+02	8.14E+01	-2.86E+01	1.35E+02	-3.97E+01	3.66E+02	1.96E+02
451	2.48E+02	8.02E+01	-3.11E+01	1.35E+02	-4.05E+01	3.55E+02	1.94E+02
452	2.47E+02	8.01E+01	-2.96E+01	1.35E+02	-4.11E+01	3.44E+02	1.91E+02
453	2.46E+02	7.88E+01	-3.00E+01	1.33E+02	-4.02E+01	3.33E+02	1.87E+02
454	2.46E+02	7.78E+01	-3.06E+01	1.32E+02	-3.95E+01	3.22E+02	1.83E+02
455	2.46E+02	7.82E+01	-3.09E+01	1.33E+02	-4.26E+01	3.13E+02	1.83E+02
456	2.44E+02	7.73E+01	-3.15E+01	1.31E+02	-4.48E+01	3.03E+02	1.79E+02
457	2.46E+02	7.68E+01	-3.17E+01	1.32E+02	-4.10E+01	2.94E+02	1.73E+02
458	2.48E+02	7.83E+01	-2.91E+01	1.33E+02	-3.92E+01	2.86E+02	1.72E+02
459	2.47E+02	7.81E+01	-2.97E+01	1.32E+02	-4.16E+01	2.77E+02	1.68E+02
460	2.45E+02	7.66E+01	-3.05E+01	1.30E+02	-3.99E+01	2.68E+02	1.67E+02
461	2.44E+02	7.57E+01	-3.16E+01	1.29E+02	-4.19E+01	2.60E+02	1.63E+02
462	2.45E+02	7.59E+01	-3.13E+01	1.30E+02	-4.03E+01	2.52E+02	1.61E+02
463	2.44E+02	7.51E+01	-3.13E+01	1.28E+02	-3.89E+01	2.44E+02	1.57E+02
464	2.46E+02	7.50E+01	-3.08E+01	1.29E+02	-3.84E+01	2.38E+02	1.56E+02
465	2.44E+02	7.41E+01	-3.09E+01	1.28E+02	-3.89E+01	2.30E+02	1.54E+02
466	2.43E+02	7.36E+01	-3.12E+01	1.27E+02	-4.00E+01	2.23E+02	1.50E+02
467	2.43E+02	7.32E+01	-3.09E+01	1.27E+02	-3.97E+01	2.17E+02	1.47E+02
468	2.43E+02	7.23E+01	-3.26E+01	1.26E+02	-3.77E+01	2.10E+02	1.47E+02
469	2.44E+02	7.32E+01	-3.22E+01	1.27E+02	-3.70E+01	2.05E+02	1.46E+02
470	2.41E+02	7.18E+01	-3.30E+01	1.25E+02	-3.97E+01	1.98E+02	1.43E+02
471	2.41E+02	7.07E+01	-3.38E+01	1.25E+02	-3.96E+01	1.92E+02	1.41E+02
472	2.42E+02	7.10E+01	-3.33E+01	1.25E+02	-3.80E+01	1.87E+02	1.40E+02
473	2.42E+02	7.07E+01	-3.42E+01	1.26E+02	-3.77E+01	1.83E+02	1.37E+02
474	2.39E+02	6.98E+01	-3.40E+01	1.23E+02	-4.10E+01	1.77E+02	1.34E+02
475	2.39E+02	6.98E+01	-3.25E+01	1.23E+02	-3.96E+01	1.72E+02	1.33E+02
476	2.38E+02	6.86E+01	-3.40E+01	1.22E+02	-4.03E+01	1.67E+02	1.32E+02
477	2.39E+02	6.94E+01	-3.33E+01	1.22E+02	-3.86E+01	1.63E+02	1.30E+02
478	2.40E+02	6.83E+01	-3.49E+01	1.22E+02	-3.73E+01	1.59E+02	1.27E+02
479	2.39E+02	6.79E+01	-3.42E+01	1.21E+02	-3.71E+01	1.55E+02	1.26E+02
480	2.38E+02	6.75E+01	-3.42E+01	1.21E+02	-3.83E+01	1.50E+02	1.25E+02
481	2.38E+02	6.78E+01	-3.47E+01	1.21E+02	-3.72E+01	1.47E+02	1.23E+02
482	2.39E+02	6.89E+01	-3.36E+01	1.23E+02	-3.59E+01	1.45E+02	1.25E+02
483	2.39E+02	6.73E+01	-3.49E+01	1.22E+02	-3.62E+01	1.41E+02	1.23E+02
484	2.38E+02	6.69E+01	-3.59E+01	1.21E+02	-3.65E+01	1.37E+02	1.21E+02
485	2.37E+02	6.56E+01	-3.51E+01	1.19E+02	-3.83E+01	1.33E+02	1.18E+02
486	2.40E+02	6.68E+01	-3.38E+01	1.21E+02	-3.48E+01	1.31E+02	1.19E+02
487	2.38E+02	6.61E+01	-3.44E+01	1.20E+02	-3.72E+01	1.28E+02	1.18E+02
488	2.37E+02	6.52E+01	-3.57E+01	1.19E+02	-3.76E+01	1.24E+02	1.16E+02
489	2.39E+02	6.60E+01	-3.46E+01	1.21E+02	-3.69E+01	1.22E+02	1.16E+02
490	2.39E+02	6.58E+01	-3.40E+01	1.20E+02	-3.36E+01	1.19E+02	1.16E+02
491	2.38E+02	6.57E+01	-3.42E+01	1.20E+02	-3.49E+01	1.17E+02	1.17E+02
492	2.37E+02	6.57E+01	-3.42E+01	1.19E+02	-3.51E+01	1.14E+02	1.17E+02
493	2.36E+02	6.51E+01	-3.50E+01	1.19E+02	-3.67E+01	1.11E+02	1.15E+02
494	2.37E+02	6.57E+01	-3.45E+01	1.20E+02	-3.54E+01	1.09E+02	1.16E+02
495	2.38E+02	6.59E+01	-3.36E+01	1.20E+02	-3.36E+01	1.08E+02	1.15E+02
496	2.37E+02	6.55E+01	-3.32E+01	1.19E+02	-3.40E+01	1.06E+02	1.14E+02
497	2.38E+02	6.59E+01	-3.38E+01	1.20E+02	-3.39E+01	1.04E+02	1.14E+02
498	2.38E+02	6.62E+01	-3.32E+01	1.21E+02	-3.40E+01	1.02E+02	1.14E+02
499	2.38E+02	6.59E+01	-3.31E+01	1.20E+02	-3.34E+01	1.01E+02	1.14E+02
500	2.37E+02	6.58E+01	-3.33E+01	1.19E+02	-3.36E+01	9.96E+01	1.14E+02

Table S4. The $pL_{0.5}$ parameter for gramibactin, EDTA, and relevant phytosiderophores calculated at different pH values, determined using thermodynamic data reported in literature.

	pH								
	**2.5**	**3.5**	**4.5**	**5.5**	**6.5**	**7.4**	**8.1**	**9.5**	**10.5**
**gramibactin**	12.9	15.1	16.6	17.0	16.4	15.5	14.4	10.7	7.8
**EDTA**[a]	12.8	14.2	14.9	15.0	14.6	13.9	13.3	10.8	8.5
**mugineic acid**[b]	3.6	5.3	6.1	6.2	6.2	6.0	5.3	1.7	0.0
**nicotianamine**[c]	0.9	3.2	5.1	6.2	7.1	7.5	7.1	4.3	1.0

[a] Ref. [9]

[b] Ref. [13]

[c] Ref. [14]

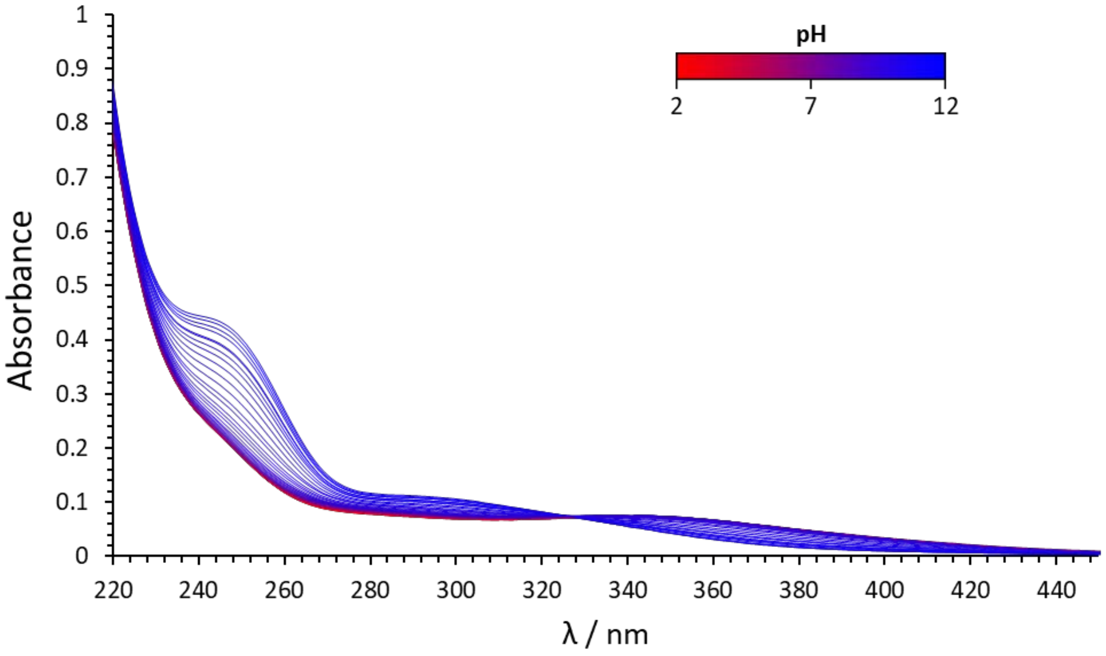

Figure S1. Experimental absorption spectra of Fe^{3+}/GBT system at 1:1.5 ratio, measured at different pH values, c_{GBT} = 0.7 mmol dm^{-3}, in I = 0.1 M KCl and T = 298.15 K.

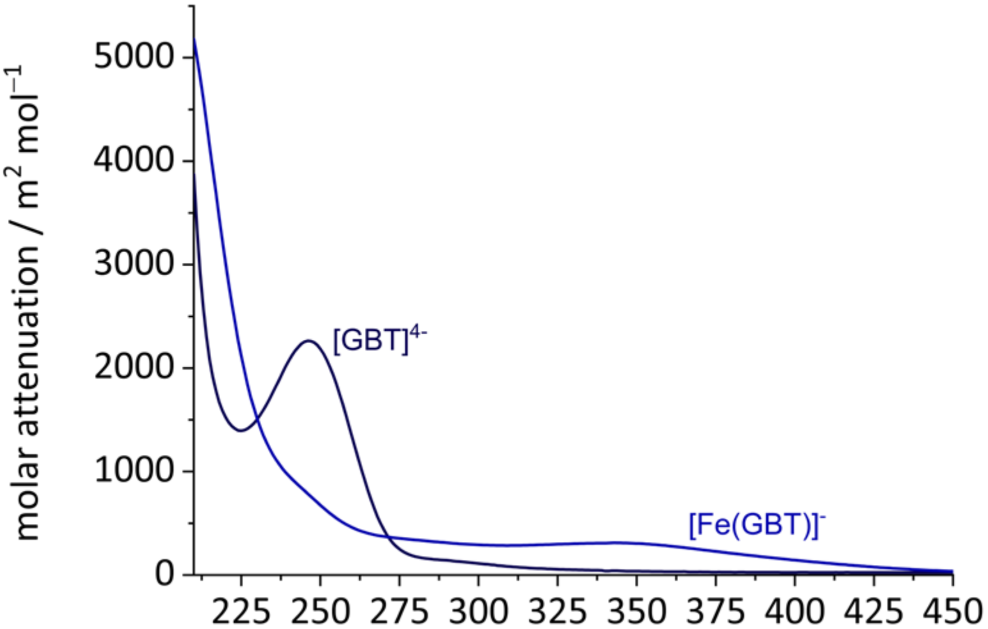

Figure S2. Comparison between the calculated molar absorvance spectra for the species $[GBT]^{4-}$ and $[Fe(GBT)]^{-}$.

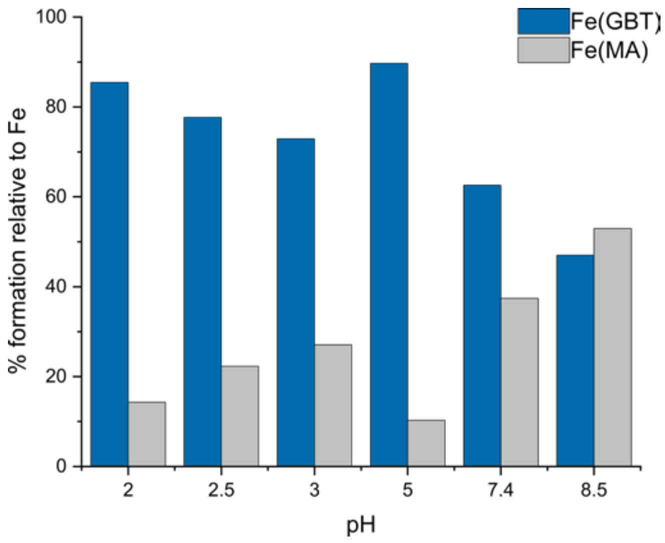

Figure S3. Percentage of [Fe(GBT)] and [Fe(MA)] species at different pH values, calculated for the Fe^{3+}/MA/GBT system considering $c_{GBT} = c_{Fe3+} = 10^{-8}$ mol dm^{-3} and $c_{MA} = 1$ mol dm^{-3}. Charges are omitted for simplicity.

Critical assessment of the parameters frequently used to quantify sequestration ability (educational tutorial):

In this context, two parameters are worth being considered, which are the pM and $pL_{0.5}$.

The former parameter has been widely used since its introduction by Raymond[15] to quantify the ability of a ligand to sequester a particular metal ion. This is mainly due to its intuitive definition (pM = –log[M]), as this value directly indicates the concentration of the free metal ion in question. However, this comes at the expense of significant drawbacks which are related to the highly susceptible misuse of this parameter. Originally, the pM parameter was defined for the case of iron(III) and, in order to be reliably determined, requires compliance with specific rules/conditions ($c_L/c_M = 10$, $c_M = 10^{-6}$ mol dm^{-3}, pH = 7.4). Not adhering to this essential prerequisite leads to inconsistency of numbers and, therefore, prevents their use for comparative assessment of the sequestration ability of different ligands.

For the pM value to correctly reflect the sequestering ability of a given ligand toward the cation under study, the latter should not undergo any competing or non-negligible reactions with all other ligands (including water, i.e., hydrolysis). This can be easily illustrated by considering the general mass balance equations of a metal ion expressed for two different ligands L and L' [Equations (1) and (2)], whose affinity toward M should be compared on the basis of pM.

$$c_{\mathrm{M}} = [\mathrm{M}] + \underbrace{\sum[\mathrm{M_pL_qH_r}]}_{a} + \underbrace{\sum[\mathrm{M_{p'}(OH)_{r'}}]}_{b} + \underbrace{\sum[\mathrm{M_{p''}L''_{q''}H_{r''}}]}_{c} + \cdots \quad (1)$$

$$c_{\mathrm{M}} = [\mathrm{M}] + \underbrace{\sum[\mathrm{M_pL'_qH_r}]}_{a'} + \underbrace{\sum[\mathrm{M_{p'}(OH)_{r'}}]}_{b'} + \underbrace{\sum[\mathrm{M_{p''}L''_{q''}H_{r''}}]}_{c'} + \cdots \quad (2)$$

In order for the pM value to effectively reflect the higher or lower affinity of L and L' toward M, the following conditions must be fulfilled:

(1) The concentration of the metal ion c_M (as well as the ligand concentration c_L) used in the calculations for both cases L and L' must be the same (as obvious and recommended by Raymond, though this basic rule is not always respected).

(2) Since hydrolysis (i.e., water or hydroxide as competing ligand) can usually not be neglected, block b should be at least numerically equal to block b'.

(3) No other competing ligands should be present and included in the calculations (i.e., $c_L'' = 0$), so that blocks c and c' can be neglected.

The only exception to the latter point is given when the contributions of blocks c and c' in both Equations (1) and (2) are numerically equal, which would require the same analytical concentration and formation of the same complexes with the same stability constants in both cases. Similarly, in relation to the second point, in order to achieve blocks b and b' to be equal, cation hydrolysis must be the same (or null) for both cases. This is one of the reasons why pM calculations performed on different cations (undergoing different hydrolysis) must be accurately evaluated, and/or why the pH value used to calculate pM must be fixed (e.g., pH = 7.4 proposed by Raymond). This last aspect is particularly relevant for pM calculations performed on ligands for which complexation is not strong enough to totally inhibit hydrolysis processes and/or when the latter processes are of comparable strength with that of the ligand complexation. In fact, it may happen (and this is more frequent than one would expect) that cation hydrolysis is strong enough to compete significantly with the complexation of the ligands, which could ultimately make the pM values of two or more ligands appear identical, although their binding capacity is different. Let us consider two cases as a simple example: for ligand L, 80% of the metal ions are complexed and 19% hydrolyzed, while for the ligand L', 60% of the metal ions are complexed and 39% are hydrolyzed. In both cases, 1% of the metal ions would remain free, which would result in the same pM value for both ligands. But, evidently, the sequestering ability of L would be 20% stronger than that of L'. Finally, it should also be mentioned the fact that all the above considerations also apply when pM calculations are performed using conditional (as originally defined by Schwarzenbach[16])

and/or semi-conditional (such as some K_f frequently used for these purposes[17]) constants. In fact, in the latter cases, although the competing reactions (including hydrolysis) are not explicitly taken into account in the calculations by using their relative equilibrium constants, in practice these reactions occur independently and they are implicitly included in the numerical value of the (semi-) conditional constants.

In order to overcome these drawbacks associated with pM, the parameter $pL_{0.5}$ has been introduced. In contrast to the pM value, the semiempirical parameter $pL_{0.5}$ does not refer to the concentration of the free metal ion, but rather represents the total ligand concentration required to sequester 50% of the metal cation (present as trace: generally a value as low as the machine limit to avoid calculation overflow should be used; in the current case this was a value of 10^{-36}) under the given conditions of the system investigated.[18]

The basic idea behind the $pL_{0.5}$ is to provide a parameter that is easy to use and calculate, which represents the effective sequestering ability of a ligand. Moreover, the $pL_{0.5}$ parameter is conceived in a way that it can be used for all kind of possible assessments.

In particular, this includes the comparison of systems with:[18]

- (i) different speciation schemes,
- (ii) different ligands toward the same metal cation,
- (iii) the same ligand toward different cations,
- (iv) the same ligand/cation system under different conditions of pH, temperature, and ionic strength,
- (v) the presence of other cations and ligands undergoing many different competing equilibria.

As a matter of fact, especially the latter point generally occurs in real systems which makes $pL_{0.5}$ an versatile parameter for the practical use in complex biological systems. The easy of application for such systems is further facilitated by the analogy with the pM value in its intuitive use in terms of greater values of $pL_{0.5}$ are associated with stronger sequestering ability of the ligand.

References

[1] Y. K. S. Murthy, J. E. Thiemann, C. Coronelli, P. Sensi, *Nature* **1966**, *211*, 1198.

[2] S. Tamura, A. Murayama, K. Hata, *Agric. Biol. Chem.* **1967**, *31*, 758-759.

[3] A. Murayama, K. Hata, S. Tamura, *Agric. Biol. Chem.* **1969**, *33*, 1599-1605.

[4] H. Iinuma, S. Kondo, T. Takeuchi, H. Umezawa, *Agric. Biol. Chem.* **1974**, *38*, 2093-2097.

[5] L. A. Dolak, T. M. Castle, B. R. Hannon, A. D. Argoudelis, F. Reusser, *J. Antibiot.* **1983**, *36*, 1425-1430.

[6] M. Nishio, M. Hasegawa, K. Suzuki, Y. Sawada, D. J. Hook, T. Oki, *J. Antibiot.* **1993**, *46*, 193-195.

[7] C. Jenul, S. Sieber, C. Daeppen, A. Mathew, M. Lardi, G. Pessi, D. Hoepfner, M. Neuburger, A. Linden, K. Gademann, L. Eberl, *Nat. Commun.* **2018**, *9*, 1297.

[8] O. V. Kovalchukova, N. Namichemazi, A. S. Bostanabad, A. I. Stash, S. B. Strashnova, I. N. Zuzin, *Russ. J. Inorg. Chem.* **2016**, *61*, 718-725.

[9] R. Delgado, M. D. Figueira, S. Quintino, *Talanta* **1997**, *45*, 451-462.

[10] C. F. Baes, *The hydrolysis of cations*, Wiley, New York, **1976**.

[11] X. W. Liu, F. J. Millero, *Geochim. Cosmochim. Acta* **1999**, *63*, 3487-3497.

[12] P. G. Daniele, C. Rigano, S. Sammartano, V. Zelano, *Talanta* **1994**, *41*, 1577-1582.

[13] M. Tasuku, I. Kunio, H. Minato, K. Shigeru, T. Sei-ichi, *Chem. Lett.* **1989**, *18*, 2137-2140.

[14] N. von Wirén, S. Klair, S. Bansal, J.-F. Briat, H. Khodr, T. Shioiri, R. A. Leigh, R. C. Hider, *Plant Physiol.* **1999**, *119*, 1107-1114.

[15] W. R. Harris, C. J. Carrano, K. N. Raymond, *J. Am. Chem. Soc.* **1979**, *101*, 2722–2727.

[16] G. Schwarzenbach, *Complexometric titrations*, Methuen & Co., Ltd, London, **1957**.

[17] R. C. Hider, X. Kong, *Nat. Prod. Rep.* **2010**, *27*, 637-657.

[18] F. Crea, C. De Stefano, C. Foti, D. Milea, S. Sammartano, *Curr. Med. Chem.* **2014**, *21*, 3819-3836.
